# Landscape of adenosine-to-inosine RNA recoding across human tissues

**DOI:** 10.1038/s41467-022-28841-4

**Published:** 2022-03-04

**Authors:** Orshay Gabay, Yoav Shoshan, Eli Kopel, Udi Ben-Zvi, Tomer D. Mann, Noam Bressler, Roni Cohen‐Fultheim, Amos A. Schaffer, Shalom Hillel Roth, Ziv Tzur, Erez Y. Levanon, Eli Eisenberg

**Affiliations:** 1 The Mina and Everard Goodman Faculty of Life Sciences, Bar-Ilan University, Ramat Gan, 5290002 Israel; 2 Raymond and Beverly Sackler School of Physics and Astronomy and Sagol School of Neuroscience, Tel Aviv University, Tel Aviv, 6997801 Israel; 3 Tel Aviv Sourasky Medical Center and Sackler school of medicine, Tel Aviv University, Tel Aviv, Israel; 4 The Institute of Nanotechnology and Advanced Materials, Bar‐Ilan University, Ramat Gan, 5290002 Israel

**Keywords:** RNA, Transcriptomics, Transcriptomics

## Abstract

RNA editing by adenosine deaminases changes the information encoded in the mRNA from its genomic blueprint. Editing of protein-coding sequences can introduce novel, functionally distinct, protein isoforms and diversify the proteome. The functional importance of a few recoding sites has been appreciated for decades. However, systematic methods to uncover these sites perform poorly, and the full repertoire of recoding in human and other mammals is unknown. Here we present a new detection approach, and analyze 9125 GTEx RNA-seq samples, to produce a highly-accurate atlas of 1517 editing sites within the coding region and their editing levels across human tissues. Single-cell RNA-seq data shows protein recoding contributes to the variability across cell subpopulations. Most highly edited sites are evolutionary conserved in non-primate mammals, attesting for adaptation. This comprehensive set can facilitate understanding of the role of recoding in human physiology and diseases.

## Abstract (web summary)

Gabay et al. provide a highly-accurate atlas of recoding by A-to-I RNA editing in human, profiled across tissues and cell subpopulations. Most highly edited sites are evolutionary conserved in non-primate mammals, attesting for adaptation.

## Introduction

Adenosine-to-inosine (A-to-I) RNA editing, catalyzed by the ADAR (adenosine deaminases acting on RNA) family of enzymes^1–4^, is the most common form of RNA editing among animals^5^. As the translation machinery largely interprets inosines as guanosines^6^ editing within the coding sequence (CDS) may lead to amino-acid substitution (“recoding”) and diversify the proteome. Recoding of GRIA2 (Glutamate Ionotropic Receptor AMPA Type Subunit 2) Q/R site is essential in mammals^7^, and recoding of a few additional well-studied targets has been shown to be functionally important. Notable examples are the antizyme inhibitor 1 (AZIN1) ADAR1-dependent recoding that promotes cell proliferation and contributes to cancer progression^8^, recoding of NEIL1 (Nei Like DNA Glycosylase 1) that results in a 30-fold reduction in the thymine glycol cleavage rate when acting on duplex DNA^9^, and FLNA (Filamin A) recoding which regulates vascular contraction and diastolic blood pressure^10^. Despite the potential importance demonstrated by these few examples, the full repertoire of human recoding sites is not known, and the scope of its functionality is still unclear.

**Fig. 1:**
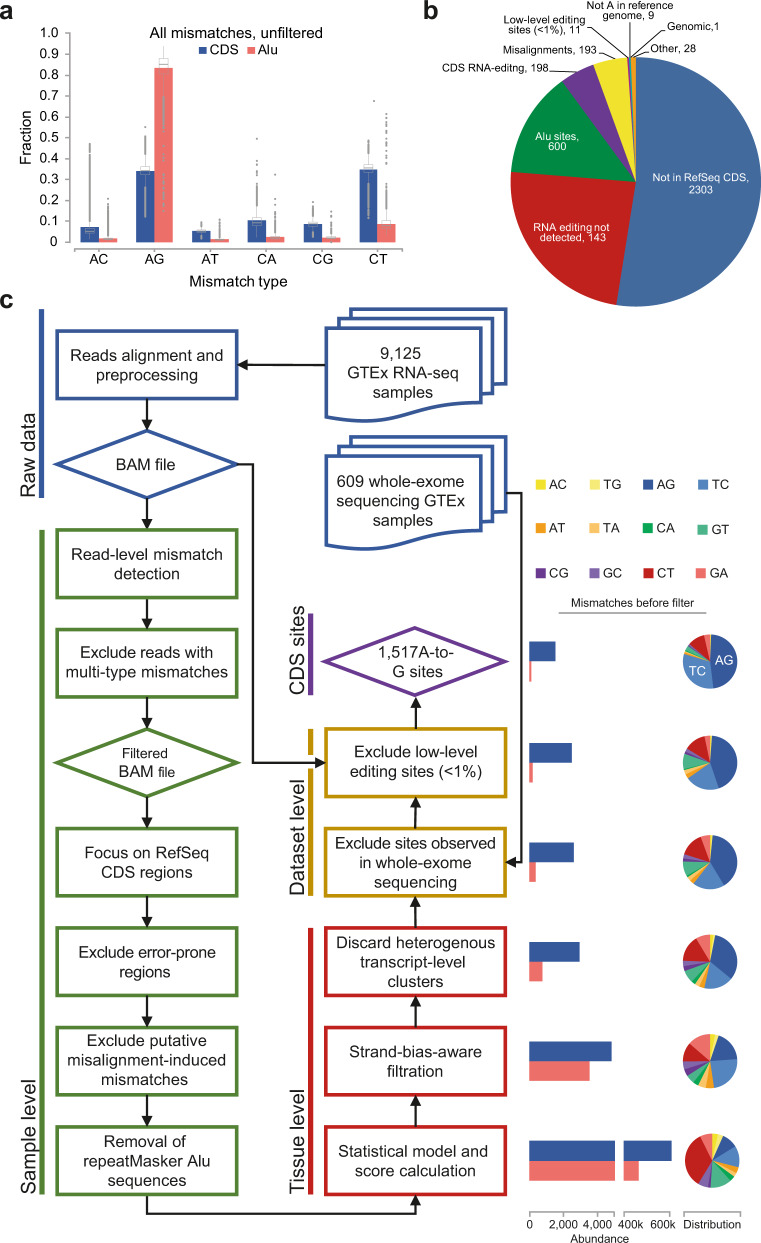
A high-precision CDS-focused RNA editing detection pipeline. **a** Relative abundance of all six types of RNA-DNA mismatches (strand-insensitive, i.e., A-to-C includes also T-to-G, etc.) following an alignment of 9125 GTEx RNA-seq samples to the reference human genome. All mismatch events are included, with no filtering. Multiple mismatches to the same genomic position are counted as separate events. Enrichment of A-to-G mismatches, presumably due to A-to-I RNA editing events is readily detectable within *Alu* elements. However, no such enrichment is observed in *Alu*-free CDS, where the editing signal is dwarfed by the noise. Colored bars and box-and-whisker plots represent the mean and the full distribution, respectively, of the relative abundances. See Supplementary Data 1 for the number of biologically independent samples per tissue. **b** Classification of all A-to-I sites annotated in REDIportal^12^ database as CDS. Merely 198 out of 4386 sites (4.5%) were detected by our pipeline as reliable CDS RNA editing sites while the majority of the sites were excluded from our analysis due to the reasons indicated in the panel. **c** A flowchart summarizing the main steps of our CDS RNA editing detection pipeline. Briefly, 9125 GTEx RNA-seq samples from various donors and tissues were aligned to the reference genome and DNA–RNA mismatches were detected and filtered within each sample separately. Results were aggregated for each tissue type for further filtration steps. Finally, the resulting candidate sites were filtered using global dataset criteria to yield a final 1517 reliable CDS A-to-I RNA editing sites (see “Methods” for details). Rightmost panel shows the mismatches abundance and distribution before each of the final filtering steps, demonstrating the increase in signal-to-noise ratio per step. Source data are provided as a Source Data file.

As part of standard sequencing protocols, inosines in the RNA are reverse-transcribed into guanosines in the cDNA. Thus, aligning the sequencing reads to the reference genome, A-to-I editing is manifested as A-to-G genome-read mismatches. Typically, recoding activity is dwarfed by A-to-I editing events at millions of sites within non-coding regions^5^, and is therefore much more difficult to detect (Fig. 1a). As a result, systematic analyses of mismatches yielded high-specificity identification of sites in human^5,11–17^, virtually all of them within *Alu* repeats, but performed poorly in coding regions. The relatively small number of recoding sites^14,18^ is overshadowed by additional sources of discrepancies between the reads and the reference genome. First, genomic variability between the reference genome and the sampled individuals translates into mismatches between the reference genome and the RNA sequenced from these individuals. Over 500 million human single-nucleotide-polymorphisms (SNPs) have been identified so far, of which almost all are rare. A typical individual genome includes tens of thousands of rare polymorphisms^19^. Matching DNA-seq data could be used to filter out these sites per sample, but it is usually not available^20^. Furthermore, even when available, somatic genomic mutations may result in mismatches misidentified as editing events. Second, systematic misalignment of RNA-seq reads to the wrong (but homologous) genomic locus could lead to an apparently consistent mismatch, to be misidentified as an editing event^21–23^. These are common in the CDS as many proteins has closely-related paralogs, polymorphic duplications, and processed pseudogenes. These mapping problems are enhanced by splicing (especially when genomic polymorphisms reside in proximity to canonical splicing junctions)^21,22^. Moreover, each individual genome harbors several megabases of non-canonical segments^24,25^, which commonly include unique duplications and processed pseudogenes. Chimeric sequences and spontaneous deamination contribute to the observed mismatches as well. Finally, technical sequencing errors are customarily cleaned using the quality score. However, the quality score seems to underestimate the error rate in specific locations such as homopolymeric sequences^26^ or reads’ ends, as well as the vicinity of mispriming events by the random hexamer primers^27^. Thus, to date, the low specificity and sensitivity of RNA-editing site detection within the coding sequence^28^ hinder global functional analyses and evolutionary studies of recoding in human.

Here we present a novel *de-novo* RNA editing detection approach, dedicated and optimized for the coding region. Analyzing 9125 human RNA-seq samples from various tissues (GTEx data)^29^, we produce a set of 1517 A-to-I RNA editing sites within protein-coding regions, with an estimated false positive rate of only 8%. Surprisingly, recoding is found in most tissues at similar levels and is not enriched specifically in the brain. There is a wide range of editing efficiencies, and a few target sites account for the majority of recoding activity. Yet, the variation of the editing level per-site across individuals is generally low, implying regulation. Most strongly-edited sites are conserved across mammals, and thus recoding as a whole seems to be under positive selection. Unexpectedly, we find a number of large clusters of recoding sites, which could lead to an extensive combinatorial diversity of the affected transcripts. Analyzing mass-spectrometry data, we provide evidence that editing at these sites results in modified proteins. Finally, we show many recoding sites to be differentially edited in cancer and pneumonia, suggesting possible etiological relevance.

## Results

### A new pipeline for detecting editing in the coding region

Previous searches and existing detection tools (e.g., SPRINT^30^, REDItools^31^, or RNAEditor^32^) for editing in human coding regions have resulted in low sensitivity and precision^27^ (Fig. 1b and Supplementary Fig. 1), due to the abovementioned difficulties. Precision has been much improved using a more stringent alignment scheme^33^. However, this approach is highly time-consuming and is not applicable for large datasets such as GTEx. Here we present a detection pipeline designed specifically to overcome these issues (Fig. 1c and “Methods”). Briefly, we focused on non-repetitive protein-coding regions, excluding, in particular, the editing-rich *Alu* exons, and analyzed 9125 RNA-seq normal human samples from the GTEx project (548 donors, 47 tissue types; Supplementary Data 1)^34^. We applied strict alignment procedures, discarding mismatches that are likely to be explained by systematic alignment or sequencing errors (Supplementary Fig. 2), and a statistical model that integrates the cumulative profile of mismatches found for all donors of a given tissue type into a single score. Using this score, we filtered out mismatches found in a small number of donors, possibly due to rare SNPs and duplications. We masked genomic loci where multiple closely-located mismatches of different types were observed, as they are suspected to result from misalignments, and used whole-exome-sequencing (WES) data to discard mismatches observed in genomic reads and further suppress false-positive detections. The breadth and depth of GTEx data allow for a reliable and consistent detection of low-level editing, as well as detection in tissues in which editing has not been studied so far. However, sites that are rarely edited (<1% editing level in all tissue types) were discarded, as they are harder to detected reliably and less likely to be functionally relevant.

**Fig. 2:**
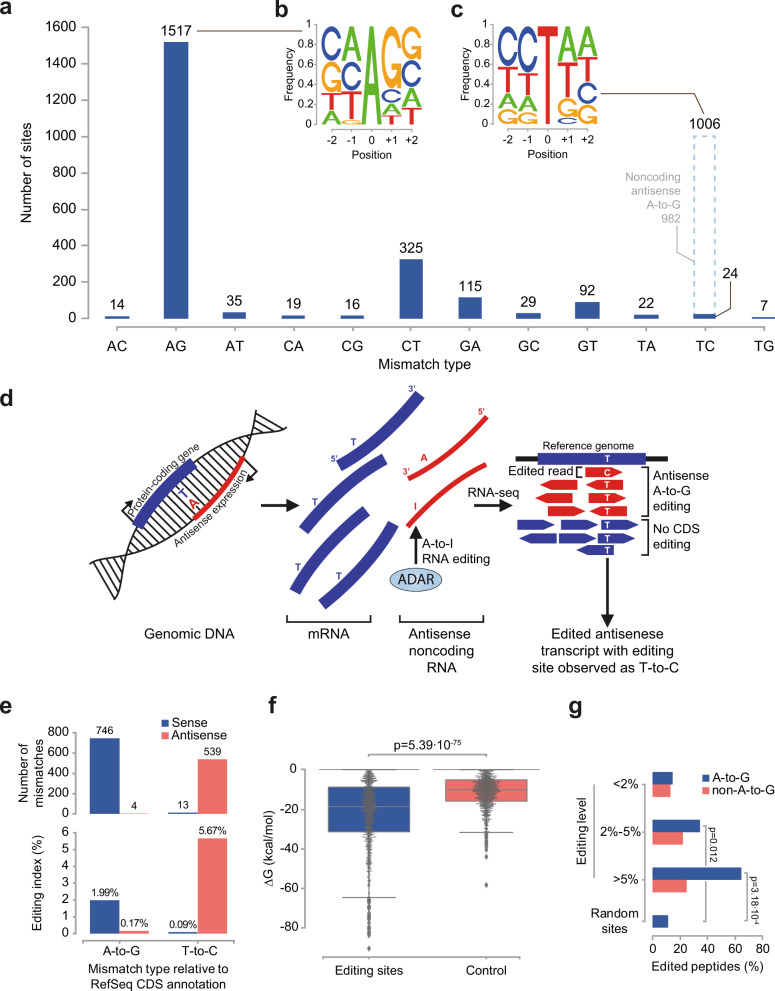
The set of 1517 detected sites exhibits ADAR-dependent RNA editing features. **a** Distribution of the 12 possible substitution types. The ADAR-derived A-to-G substitution constitutes ~68% of the total mismatches detected within the coding sequence. We estimate that ~982 of the 1006 T-to-C sites (~98%; dashed light-blue line) are due to non-CDS A-to-I editing events on the non-coding strand (see panel E below). **b** The local DNA sequence preference surrounding the A-to-G sites is consistent with the previously-reported ADAR preference. **c** The T-to-C sites show the same local sequence preference, reverse-complemented, suggesting that they result from ADAR-mediated editing of RNA expressed from the antisense strand. **d** An antisense RNA (red) expressed from a locus that overlaps a protein-coding exon (blue). A-to-I editing produces inosines in the antisense transcripts, manifested as Gs in RNA-seq reads. Mapping these strand-insensitive reads to the reference genome, antisense editing events appear as T-to-C mismatches with respect to the protein-coding strand. **e** Top: Analysis of strand-specific RNA-seq samples reveals that 539 out of the 552 T-to-C sites covered by the strand-specific RNA-seq dataset (~98%) were actually A-to-G substitutions on the antisense strand. In comparison, 99.5% of sites annotated as A-to-G originated from the coding strand, as expected. Bottom: The strand-specific RNA editing index (“Methods”) was calculated separately for A-to-G and T-to-C mismatches, showing negligible, indistinguishable from zero, editing level for T-to-C sites on the coding strand. **f** A swarm plot showing the distribution of free energies for in*-*silico RNA secondary structures surrounding the 1517 A-to-G sites and the same number of random adenosines as controls. The A-to-G editing sites form significantly more stable structures. *P*-value by Mann–Whitney test. Box-and-whisker plots show the medians (horizontal lines), upper and lower quartiles (box edges), and 1.5 × the interquartile range (whiskers). **g** Proteomic mass spectrometry data (“Methods”) reveals peptides supporting editing for 65% (11/17) of the sites edited to >5% and covered by peptides, compared to 11% (18/158) for random control sites (*p* = 0.00032; two-tailed Fisher’s exact test). In comparison, peptides supporting editing were found for only 15% (13/89) of the weakly-edited (<2%) sites (*p* = 0.56, compared to random control sites).

This pipeline resulted in a reliable set of 1517 human CDS A-to-I RNA sites (Fig. 2a and Supplementary Data 2), showing the familiar 5′ and 3′ neighbor-sequence preferences of mammalian ADARs^35^ (Fig. 2b). The large number of T-to-C sites (1006) also exhibit the familiar (reverse-complement) motif (Fig. 2c), suggesting that they are mostly due to A-to-I editing of RNA molecules transcribed from the antisense strand, complementary to the annotated coding RefSeq exons (Fig. 2d). To verify that, we analyzed a strand-specific RNA-seq dataset^36^, for which one can separate the reads based on their genomic strand of origin. As expected, the T-to-C signal observed in GTEx non-stranded data does not show up in reads originating from the coding strand and is fully accounted for by A-to-G editing of reads transcribed from the non-coding strand (Fig. 2e). Based on these results, we estimate that only ~24 of the 1006 T-to-C sites observed are false-positive detections (“Methods”). Note that the editing levels found at the detected T-to-C sites using stranded data are ~3-fold higher than the A-to-G sites, due to a lower detection power in the antisense strand. Expression of this strand is typically lower compared to the protein-coding strand (see Supplementary Data 3), and thus the editing signal is masked by the many unedited reads coming from the sense strand. Accordingly, the sites that are detected under these conditions are typically more strongly edited, and the editing levels reported for these sites based on non-stranded data are underestimated. Some of the C-to-T sites detected (mostly in blood samples, where APOBEC3A is highly expressed) may reflect DNA or RNA editing by members of the AID/APOBEC family of deaminases. Indeed, one of the detected sites is the only known C-to-U recoding site in APOB (Apolipoprotein B)^37,38^. Accordingly, we used the next abundant mismatch type, and estimated the false positive rate to be 8% (115 G-to-A sites).

ADAR enzymes bind to dsRNA secondary structures^39^. Accordingly, the predicted editing sites tend to be part of putative intra-molecular dsRNA structures (Fig. 2f and “Methods”). To test whether editing of these sites translates into novel protein isoforms, we have analyzed 311 human mass-spectrometry proteomic samples taken from the PRIDE database^40^ (Supplementary Data 4). Shotgun proteomics results in partial coverage of the tryptic peptides^41^, and thus only 138 of the sites are covered by peptides, regardless of their editing state. Of these, peptides supporting the edited version of the transcripts were observed for 35 sites (25%) (“Methods”, Fig. 2g and Supplementary Data 5). Reassuringly, edited peptides were observed for 65% (11/17) of the covered sites predicted to be appreciably edited (>5% editing in RNA-seq data).

The sensitivity of our pipeline is demonstrated by recovering all 38 previously published mammalian conserved sites^42^, except for one of the IGFBP7 (Insulin Like Growth Factor Binding Protein 7) sites^43^ that resides adjacent to a homopolymeric sequence and was, thus, filtered out. However, the overlap of our list with previously published sets of human CDS sites^12^ is rather low (Supplementary Fig. 3), which we attribute to low specificity and sensitivity of previous approaches. Indeed, for most of the previously published sites we can pinpoint the reason for their misidentification (Fig. 1b).

### Tissue-specificity of CDS editing sites

**Fig. 3:**
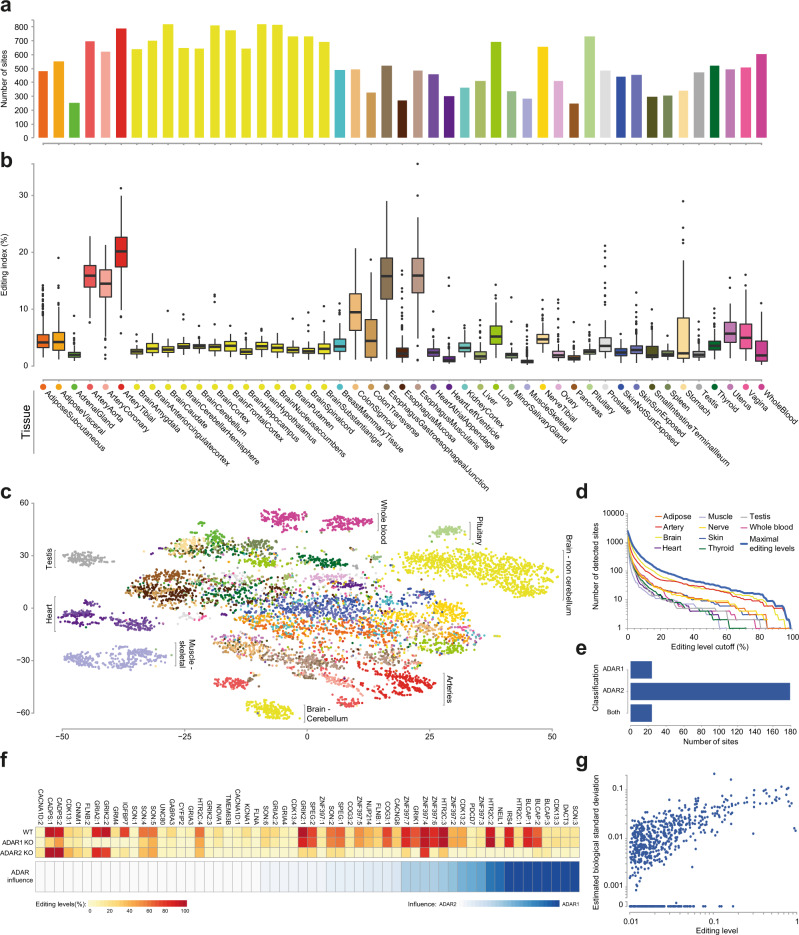
Tissue-dependence and ADAR-specificity of CDS editing sites. **a** Number of sites detected and edited to >1% per-tissue (out of the total 1517). The highest numbers of sites are observed in the nervous system, arteries, and lung. **b** Box-and-whisker plots, depicting the distribution of per-tissue CDS editing index values reveal that although the number of edited sites in the brain is large, CDS editing activity (number of deamination events) is comparable to most other tissues and is much lower than in arteries, colon or esophagus. Box-and-whisker plots show the medians (horizontal lines), upper and lower quartiles (box edges), and 1.5 × the interquartile range (whiskers). See Supplementary Data 1 for the number of biologically independent samples per tissue. **c** t-SNE^118^ dimensionality-reduction analysis (“Methods”) reveals highly distinctive CDS editing pattern in cerebellum, arteries, testis, and non-cerebral brain regions. **d** The number of sites detected depends strongly on the editing level cutoff. For all tissues, about half of the detected sites are weakly edited (<1%). **e** Analyzing previously published RNA-seq data from ADAR1- and ADAR2-overexpressing human cell lines, we classify 227 of the sites (well covered and sufficiently edited in cell lines data) based on the editing enzyme. The vast majority of these are targeted by ADAR2 (see Supplementary Data 8). (**f**) Analyzing previously published RNA-seq data from ADAR1 and ADAR2 knockout mice^14,49^, we calculate the ADAR Influence value (see Methods) for 58 sites conserved in mouse (all sites with >5% in wild type mouse and significant, Fisher *p* < 0.05, difference between wild type and the double-knockout mouse, excluding the GRIA2 site that was genomically edited to G in the double-knockout) (See Supplementary Data 9). The heatmaps depict the editing levels and the ADAR Influence value (darker: ADAR1, lighter: ADAR2). **g** The biological variability (“Methods”) in editing levels across individual donors. Many of the strongly edited sites exhibit standard deviations much smaller than the mean, implying regulation on the RNA editing activity. Source data are provided as a Source Data file.

Editing in the coding region is not specific to brain regions. The number of sites discovered per tissue is similar for arteries, lung, and brain tissues (Fig. 3a). Furthermore, quantification across tissues of the global editing levels in the coding sequence (the RNA editing index^33,44,45^, a weighted average over all sites; Methods) shows (Fig. 3b) that the arteries^10^, colon, and esophagus are edited to a much higher extent than the brain. However, most of the recoding signal in these tissues is due to a small number of targets (mainly the highly-expressed and highly-edited FLNA and IGFPB7^43^) whereas editing in the brain is much more diverse and affects a larger number of targeted genes and sites (Fig. 3a and Supplementary Fig. 4). Interestingly, distinct editing profiles are found for different tissues (e.g., blood, testis, heart and muscle) (Fig. 3c). Sites are not enriched or depleted in annotated protein domains compared to other coding regions (incidence rates 1.6 × 10^−4^ and 1.8 × 10^−4^, respectively; Fisher’s exact test *p* = 0.06, not significant). Note that the distribution of editing levels is heavily skewed towards weakly-edited sites (Fig. 3d), and thus the number of sites declared as being edited depends strongly on the editing level cutoff.

Analyzing single-cell RNA-seq data (Methods), we find editing levels to vary dramatically between different cell populations within the same tissue^46^ (Supplementary Data 6, 7). Remarkably, the variation of the editing pattern across cell populations is complex, and cannot be explained by a simple regulation mechanism. For example, editing in the brain is significantly higher for endothelial cells compared to excitatory neurons for several sites (CCNI (Cyclin I), COPA (COPI Coat Complex Subunit Alpha), and AZIN1), but lower at the TMEM63B (Transmembrane Protein 63B) site. Similarly, for many sites (GRIA2, GRIA3, TMEM63B, CYFIP2 (Cytoplasmic FMR1 Interacting Protein 2), and KCNA1 (Potassium Voltage-Gated Channel Subfamily A Member 1)) editing is significantly higher for excitatory neurons compared to oligodendrocyte precursor cells in the brain, but lower at the COPA site. We note that excitatory and inhibitory neurons exhibit markedly different editing levels at two key sites known to regulate neural activity, GRIA2 RG site (editing level 92% for excitatory neurons, compared to 61% in inhibitory neurons) and KCNA1 (69% vs. 10%). Interestingly, ADAR3 (also known as ADARB2), known to regulate RNA editing levels^14,47^, was previously implicated as one of the few genes differentiating between subpopulations of inhibitory neurons^48^.

### Validation of results in additional datasets

In order to further validate our A-to-G results, and further support our conclusion that the large number of T-to-C sites result from A-to-I editing of RNA molecules transcribed from the antisense strand we have analyzed three additional strand-specific RNA-seq datasets (see “Methods”), together with the previously mentioned dataset^36^. Looking at the coding strand, we find that 1375 of the 1517 A-to-G sites were covered in at least one of the four datasets (>100 reads). Of these, 948 were found to be edited in at least one dataset (FDR corrected binomial *p*-value < 0.05 for the pooled data, per dataset). Note that sites that did not show evidence of editing could be edited in other tissues (not present in this validation study) or missed due to a low editing level and insufficient coverage. For the 1006 T-to-C sites, 885 were covered but only 40 (4.5%) of them have passed the binomial test with *p* < 0.05. Looking at the opposite strand, only 76 of the 1517 A-to-G sites were covered in at least one of the four datasets, and only 5 of them (6.6%) have passed the binomial test with *p* < 0.05, while 582/1006 T-to-C sites were covered, and 545 of them were found to be edited. Taken together, these results demonstrate the accuracy of our set of editing sites, and the proposed explanation for the T-to-C mismatched observed in non-stranded data.

### ADAR-specificity of CDS editing sites

ADAR2 (also known as ADARB1) is the main contributor to editing in the coding sequence. Quantifying editing at our sites upon ADARs overexpression in HEK293 cell lines^14^, we were able to classify 24 sites as ADAR1-dependent, 179 sites as ADAR2-dependent, and 24 sites were shown to be edited by both (Fig. 3e and Supplementary Data 8). Of the sites whose editing is conserved in mouse, we found 11 sites in which editing disappears upon ADAR2 knockout (KO), compared to only 3 sites in ADAR1 KO. In 45/58 sites (78%), editing is suppressed more strongly upon ADAR2 KO (Fig. 3f and Supplementary Data 9)^49^.

Assuming editing in coding sequences is functionally important, we expect the editing levels to be regulated^50^. Looking at the variability of editing levels across individuals (controlling for the sampling noise; “Methods”), we find that weakly edited sites are generally noisy, with standard deviation as high as the mean. However, the strong sites mostly show a well-regulated behavior (Fig. 3g and Supplementary Data 10).

### Clustering of CDS editing sites

**Fig. 4:**
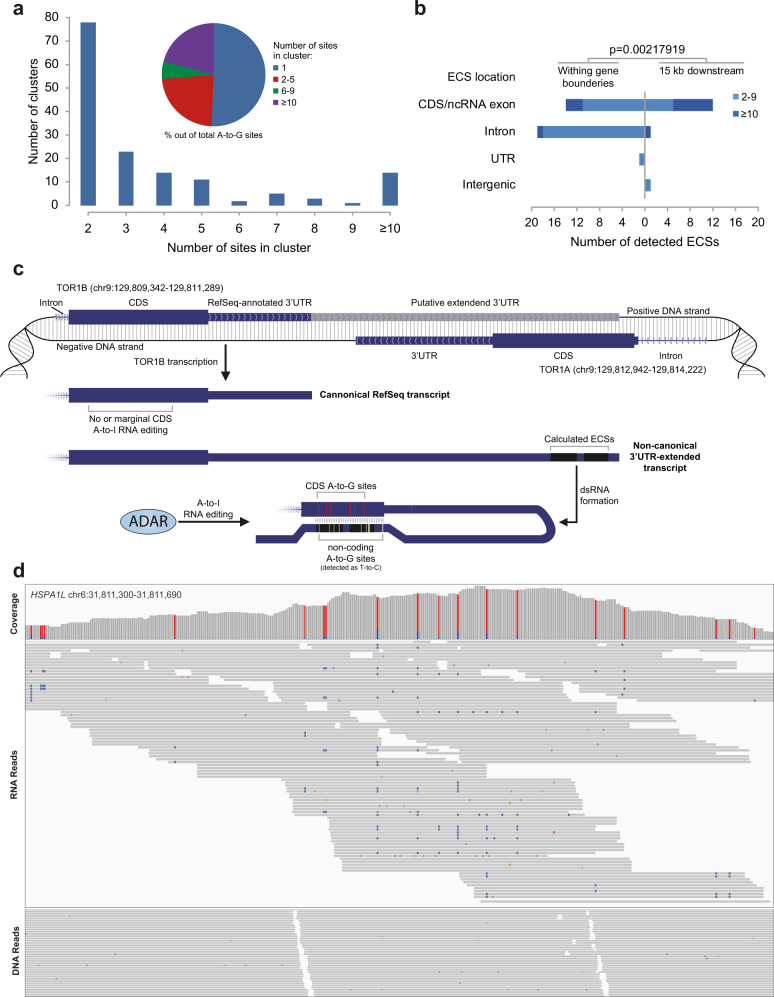
Many of the CDS editing sites are clustered. **a** Bar plot: Distribution of editing clusters by size. Pie chart: Relative abundance of editing sites by cluster size. **b** Editing requires a dsRNA secondary structure, often depending on a distant editing complementary sequence (ECS). Distribution of putative ECSs location (“Methods”) shows that smaller clusters tend to depend on an intronic ECS, while the ECS for large clusters (≥10) is often found out of the annotated gene boundaries, overlapping an exon of a neighboring downstream gene. **c** Editing due to a downstream reversely-oriented paralog gene, overlapping an extended 3′UTR. The TOR1B gene contains a 10-site RNA editing cluster in its coding sequence. The corresponding ECS is a closely-related sequence that overlaps a coding exon within the neighboring paralog TOR1A gene (genomic coordinates of the last exon of both genes are shown). Presumably, the 3′UTR of edited TOR1B transcripts is longer than that of the Refseq canonical transcript, extending to overlap the coding sequence of *Tor1a* (on the opposite strand). These extended-3′UTR variants of the *Tor1b* transcripts contain two highly-similar reversely-oriented sequences, and can form a long and stable dsRNA structure, resulting in extensive editing of both the CDS (red lines) and the TOR1A-overlapping 3′UTR (yellow lines). A 14-site cluster of T-to-C sites is detected in the coding sequence of TOR1A, a hallmark of A-to-I editing in antisense transcripts (see Fig. 2d). See Supplementary Data 3 for further evidence for expression and editing of the extended UTR of TOR1B. **d** Editing at one of the large clusters within the HSPA1L transcript. Up: pile-up of the RNA reads’ coverage, 19 different editing sites are observed in this 391 bp-long segment of the editing cluster (red and blue bars stand for A and G fractions, respectively). Bottom: Matched RNA and DNA sequences (presented is the pooled data for the three individuals, for simplicity). Editing events (A-to-G mismatches) are shown in blue (different colors represent other type of mismatches). The absence of A-to-G mismatches in the matched DNA samples and lack of linkage between neighboring sites both support the sites being RNA-edited.

It is well known that editing sites in non-coding regions of the genome are clustered^51–55^. Interestingly, we observe here clustering of many sites in the coding region. About half of the sites belong to dozens of clusters, some of which include over 10 sites each (Fig. 4a). Moreover, for many editing sites, we find a putative editing complementary sequence (ECS) that is located up to 15 kb downstream to the canonical transcript. These structures are mostly overlapping with a paralog gene expressed from the other strand and account for many of the larger clusters (Fig. 4b). We believe these events are due to an extended UTR, not included in the canonical RefSeq transcript, which are known to be expressed in brain tissues^56^. For example, an editing cluster including 19 sites is located within the coding sequence of *cldn9* (Claudin 9). A long ECS is found 1741 bp downstream, overlapping the coding sequence of a paralog gene, *cldn6* (Claudin 6). One does observe a cluster of T-to-C sites within this putative ECS, attesting for expression and editing of this region in the *CLDN9* coding strand (opposing *CLDN6* coding sequence). Consistently, T-to-C mismatches are over-represented in the brain, especially in cerebellum (Supplementary Fig. 5), where the long UTRs are expressed. This suggests a new model for editing regulation, where editing depends on the expression of the longer UTR isoform, which may be tissue-dependent (Fig. 4c). The HSPA1L (Heat shock 70 kDa protein 1-like) transcript harbors two of the largest clusters found (87 sites). Since this gene is located in vicinity of the highly polymorphic region of the HLA genes in chromosome 6, we have further validated editing in this gene by sequencing matched DNA and RNA blood samples from three individual persons (Fig. 4d; “Methods”).

Differential editing of specific recoding sites was reported in multiple diseases^57,58^. We have re-analyzed^59–61^ matched normal and cancer samples of the TCGA dataset^62^ and found eight sites from our set which show a significant and appreciable (>10%) change of their editing level in at least one of the nine cancer types studied (Supplementary Data 11). In light of the strong recoding activity in arteries, we have also looked for differential editing in the arteries using GTEx data. Interestingly, of the 27 different diseases and conditions annotated we have found significant and appreciable (>10%) differential editing only in one disease, pneumonia, for which higher editing is observed at five sites (Supplementary Data 12).

### Conservation of recoding sites, and signals of adaptation

**Fig. 5:**
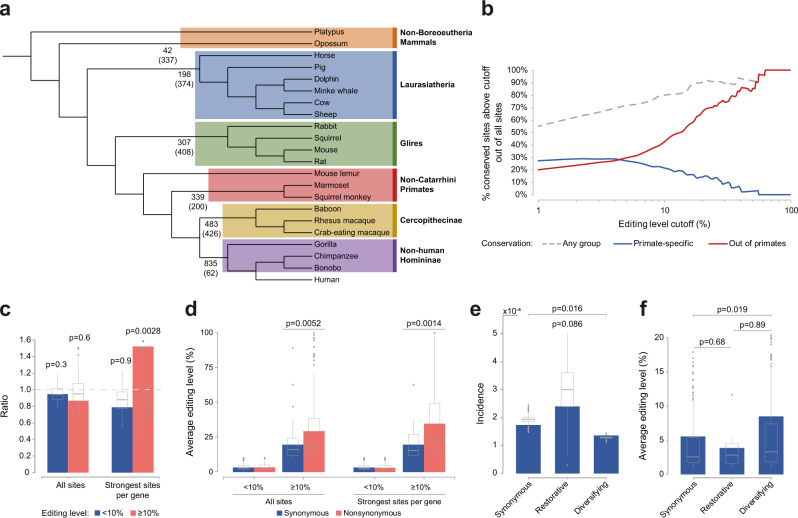
Evolution, conservation, and signs for adaptation in CDS-editing sites. **a** Phylogenetic tree of the mammalian species analyzed (branch lengths not to scale). For each of the six evolutionary groups, numbers on left present the number of sites conserved between the human and the last common ancestor (LCA) of the group, and, in parentheses, number of human sites which are determined as not edited in the group (“Methods”). **b** Fraction of evolutionary-conserved sites whose (human) editing level is above a given cutoff, as a function of this cutoff. **c** Ratio of incidence rates for synonymous and non-synonymous sites is approximately unity (dashed gray line), for weak (<10%) and strong (≥10%) sites alike. For the strongest site in the gene, weak nonsynonymous editing sites are slightly depleted while strong nonsynonymous sites are enriched, suggesting positive selection and adaptivity. Box-and-whisker plots represent the distribution of ratios over 10^6^ random As, controlling for the ±1 bp nucleotide context. **d** Mean (colored bars) and distribution (box-and whisker) of editing levels. Number of sites: 879 nonsynonymous and 378 synonymous weak sites; 177 and 83 strong sites. Considering the strongest site per gene only: 408 nonsynonymous and 211 synonymous weak sites; 105 and 28 strong sites. **e** Sites were classified into three categories based on the reconstructed amino acid encoded by ancestral primates (“Methods”): synonymous, restorative (i.e., recoding results in an amino acid encoded by ancestral primate), or diversifying sites (otherwise). The incidence rate (“Methods”) is higher for restorative editing (not significant) and slightly lower for diversifying sites, relative to synonymous ones. Box-and-whisker plots present distribution of ratios over 10^6^ random As, controlling for the ±1 bp nucleotide context in each of the three categories. **f** Mean (colored bars) and distribution (box-and whisker) of editing levels for 581 diversifying, 8 restorative, and 239 synonymous sites. Box-and-whisker plots show the medians (horizontal lines), upper and lower quartiles (box edges), and 1.5 × the interquartile range (whiskers). *P*-values by randomization test (**c**, **e**) or two-sided Mann–Whitney test (**d**, **f**). Source data are provided as a Source Data file.

To gain insight into the evolution of recoding in the mammalian lineage, we have analyzed 5673 RNA-seq samples originating from 21 non-human mammals^63–93^ (Fig. 5a; Supplementary Data 13 and “Methods”) and quantified the editing level at the one-to-one orthologous locations for each of the 1517 human-detected sites (when available). Contrary to previous estimates, we find that a sizable fraction of human sites are conserved across species. As many as 835 editing sites (~55% of the set) are conserved in at least one of the groups (Fig. 5a). The fraction of conserved sites increases with the (human) editing level (Fig. 5b). For example, 46% of sites edited to >10% and 75% of sites edited to >30% are conserved out of the primates. On the other hand, only 17 sites can be identified as (probably) human-specific (“Methods” and Supplementary Data 14).

It was previously pointed out that the reported editing sites in human coding sequences are depleted in nonsynonymous sites (ratio of nonsynonymous to synonymous editing incidence rates ${f}_{N}/{f}_{S}=0.6$) and that editing levels of nonsynonymous sites are generally lower, suggesting an overall deleterious effect^94^. Revisiting this findings with our set of coding-sequences editing sites, we observe neither depletion of nonsynonymous sites nor a lower editing level (abundance ${f}_{N}/{f}_{S}=0.93$; not significantly different from unity, *p*-value = 0.20, proportion test; editing level *p* = 0.42, Mann–Whitney test) (Fig. 5c, d). Moreover, taking into account that neighboring synonymous and non-synonymous sites are not mutually independent, and a strong adaptive nonsynonymous site may be accompanied by synonymous (and nonsynonymous) weak “satellite” sites, we looked again at the abundances of sites, focusing on strong (≥10%) sites and including only the strongest site in each gene. Here, one observes an enrichment and an increased editing level of nonsynonymous editing (${f}_{N}/{f}_{S}=1.5$; *p*-value = 0.0028, proportion test; editing level *p* = 0.0014, Mann–Whitney test) (Fig. 5c, d), indicating positive selection. Following Jiang and Zhang^95^, one may distinguish between restorative and diversifying recoding sites. In the former, recoding introduces an amino acid that was encoded in the genome of an ancestral primate species (“Methods”), whereas diversifying editing introduces a novel protein isoform within the considered phylogenetic tree. Comparing the incidence rates and the editing levels of synonymous, restorative and diversifying editing sites, one finds that although diversifying sites are slightly depleted compared to synonymous editing (incidence rate ratio 0.78, *p* = 0.016), their mean editing level is 1.5-fold higher (*p* = 0.019) (Fig. 5e, f). Taken together, these results suggest that the 260 strongly-edited (≥10%) human editing sites are overall adaptive.

### Evolution of editing sites

**Fig. 6:**
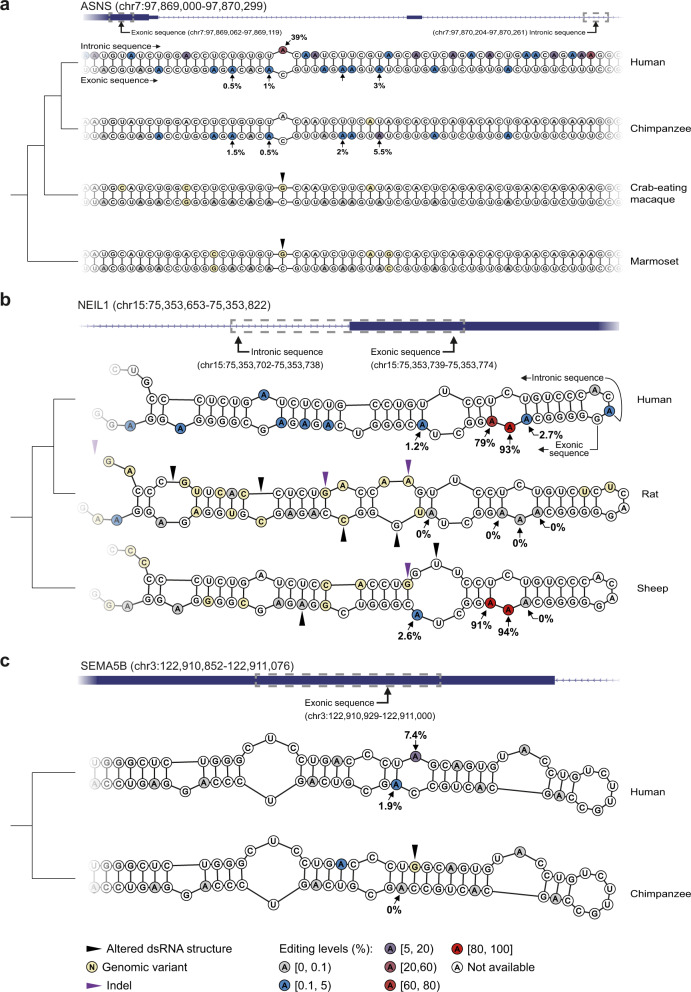
Species-specific dsRNA structures contribute to the formation of novel RNA editing sites. **a** Several hominid-specific exonic RNA editing sites were introduced to the *Asns* gene following a single G-to-A genomic substitution modifying the RNA secondary structure. Two uppermost sequences show a few CDS adenosines being edited in human and chimpanzee. These editing events are not conserved in more distant primates. In non-hominid primates (two lowermost structures), the RNA structure is very similar, except for a single hominid-specific mismatch (internal loop) feature, resulting from a genomic G-to-A substitution in the intronic ECS of hominids. **b** The extensive editing in NEIL1 CDS seen in primates (top) is conserved in some non-primates as well (bottom). However, editing is much weaker in rodents (middle). Mutations in the rat sequence alter the secondary structure, while the sheep structure is more similar to the human one. **c** The emergence of human-specific RNA-editing site. A human-specific G-to-A substitution results in an A:C mismatch within a dsRNA structure already present in the ancestral hominid. This newly introduced adenosine is efficiently deaminated by the ADAR enzymes, and an additional weaker site appears in the complementary sequence. In each of the three panels, the top track shows the locus of the edited CDS region and the ECS (gray dashed-line boxes) within the corresponding gene (blue boxes – exons; lines with arrowheads – introns); the bottom track shows the corresponding calculated secondary structures. The topology of the phylogenetic tree for the species presented is shown on the left (branch lengths not to scale). Species names are denoted on the right. Major structure alternations along with indels and single nucleotide substitutions (relative to human) are indicated. Adenosines in all structures are annotated in different colors based on their calculated RNA editing levels (pooling all brain samples available, per species; Gray – no detectable editing, blue – low editing levels; red – high editing levels). Editing levels were not assessed for sites covered by <50 reads.

The detailed comparison of human editing sites to other mammals enables us to examine possible evolutionary routes through which novel editing sites may emerge. Interestingly, even a single mutation may be responsible for the introduction of editing sites. For example, an exon of the ASNS gene pairs with an intronic sequence to form a perfectly-paired 50 bp-long dsRNA. This structure is conserved in most primates, but we find no editing (or merely the editing of a single position) out of the Homininae. A single G-to-A mutation in the intron resulted in an A:C mismatch specific to the Homininae. Such a mismatch flanked by long dsRNAs is known to be a preferred ADAR target^52^. Consistently, the mutated intronic A is strongly edited, and four addition editing sites appear in the vicinity of the exonic C (Fig. 6a). On the other hand, a few mutations can abolish strong editing sites, as is demonstrated by the NEIL1 example. Two adenosines are strongly edited (79 and 94%) in NEIL1 coding sequence in human, but not in rodents (no editing in rat; 9 and 0.5% editing in mouse). It was previously suggested that the strong editing is primate-specific and depends on a neighboring *Alu* sequence^96^. However, these two sites are extensively edited in the *Alu*-lacking sheep (91 and 94%). The secondary structure is similar in all mammals, but a few mutations result in the appearance of two asymmetric internal loops in rat (compared with one in human, and a larger symmetric loop in sheep), which may be responsible for the dramatic suppression of editing (Fig. 6b). Finally, a human specific editing site appears following a G-to-A mutation within an exon of the *sema5b* (Semaphorin 5B) gene (Fig. 6c). The mutation is located within a region that forms a dsRNA structure, and changes a G:C pair into an A:C mismatch, which is favorably edited.

## Discussion

Recoding by A-to-I editing provides an additional layer of the complex regulation of the proteome. Mapping the landscape of recoding sites enables future studies to reveal the full functional impact of recoding in mammals, across previously unstudied tissues, pathways, and pathological conditions. It also provides a better understanding of the structural and sequence determinant underlying recoding efficiency and regulation which may be then utilized for an improved design of ADAR-based RNA engineering strategies.

Most sites in our list are weakly edited. Furthermore, the number of detected sites depends strongly on the editing level cutoff, and there are thousands of additional sites edited to <1% in all tissues examined. These are less likely to be functionally important. However, it is possible that sites appearing to be weakly edited when averaged over a tissue under normal conditions, exhibit much higher editing levels under specific conditions, in specific subpopulations of cells^46^, or even at a single-cell level^97,98^. Combining large-scale single-cell sequencing data with the editing detection method presented here could reveal the full scope of recoding and its functional impact.

## Methods

### Human RNA-sequencing data

RNA-seq samples from the Genotype-Tissue Expression (GTEx) project^34^ were used as the core input for our *de-novo* RNA editing detection pipeline. Raw data (76bp-long, paired-end reads) were accessed via the database of Genotypes and Phenotypes (dbGaP; Study Accession: phs000424.v8.p2) and FASTQ files were downloaded via Sequence Read Archive (SRA) using SRA Toolkit (v2.8.0). We used the raw reads and not the alignment files available from GTEx, as they include multi-loci mapped reads, which hinder *de-novo* discovery of editing sites. A total of 9125 RNA-seq samples from 548 donors and 47 tissues were analyzed (Supplementary Data 1).

### Nonhuman RNA-sequencing data

Nonhuman mammalian RNA-seq datasets were collected from varied sources^63–93^ to test the conservation of the *de-novo* detected human editing sites across the mammalian lineage. Altogether, 5673 RNA-seq samples (Supplementary Data 13) from 21 different nonhuman species (Supplementary Data 15) were analyzed.

### Reads alignment and BAM processing

Raw reads were mapped to the reference human genome using STAR RNA-seq aligner (vSTAR_2.5.2b)^99^ as follows: The reference genome (hg38; analysis set version) was downloaded from the UCSC Genome Browser^100^ (http://hgdownload.soe.ucsc.edu/goldenPath/hg38/bigZips/analysisSet/), and indexed using default parameters and no annotation files. The reads were then aligned in a per-sample basic two-pass mode (-twopassMode Basic), keeping only uniquely mapped reads (-outFilterMultimapNmax 1). The “-outSAMattributes All” option was used in order to output the MD tag which is required for the detection of mismatches in later steps.

Duplicated reads were marked using Picard Tools MarkDuplicates program (v2.6.0-SNAPSHOT; http://broadinstitute.github.io/picard/), later to be ignored by our pipeline. Next, BamUtil clipOverlap (v1.0.13; https://github.com/statgen/bamUtil)^101^ was applied to clip one of the reads in pairs where the two pair-mates overlap. Different RNA-seq samples originated from the same donor and tissue (technical duplicates) were merged into a single BAM file using SAMtools marge (v0.1.18; http://www.htslib.org/)^101^.

Nonhuman samples were processed as described for human and were mapped to the respective reference genomes (Supplementary Data 15).

### Read-level detection of mismatches

BAM files were converted into SAM format using SAMtools view (v0.1.18), and were analyzed to detect all mismatches between the reads and the reference genome. We excluded mismatches that were located at the ends of reads or alignments (5 bp form each end) or near splice-sites (4 bp), adjacent to a homopolymeric sequence in a length of 5 bp or more (upstream or downstream), or in positions with low base quality score (*q* < 30). In addition, we excluded reads of low quality (25% of the read with *q* < 20), and those which failed the platform/vendor quality checks, were not mapped as a proper pair, were PCR or optical duplicates, or included three or more mismatches of more than two distinct types. Eventually, a list of mismatches of all twelve possible substitution types for all donors in every available tissue was obtained. For each mismatch the number of reads supporting it and the total number of covering reads (i.e., total depth) were reported. In addition, the coverage by reads mapped to the positive strand of the reference genome and the number of those reads supporting the mismatch were recorded.

### Annotation-based filtering of mismatches

Our analysis focused solely on protein-coding regions within curated transcripts, as annotated by RefSeq (records with an NM_ prefix; see below). Regions, where both strands are annotated as RefSeq coding exons (total length: 5639 bp), were discarded. As GTEx RNA-seq data is not strand-specific, we assumed the expressed strand to be the annotated coding strand. To minimize the extent of erroneously-called substitutions we excluded from the analysis the following genomic regions or locations that are suspected to introduce false-positive detections of RNA editing sites: (i) Common genomic single-nucleotide variations in dbSNP150 or dbSNP147, (ii) 50 bp regions around common insertions and deletions (indels) in dbSNP150 or dbSNP147. (iii) Rare SNPs (found in <1% of the population) where the reference genome includes the rare allele according to the alleles frequencies reported in either the 1000 genome project^102^ or the Trans-Omics for Precision Medicine (TOPMed; https://www.nhlbiwgs.org/) project using dbSNP150 and dbSNP146. In addition, “known issues” reported directly by dbSNP (https://ftp.ncbi.nih.gov/snp/organisms/human_9606_b150_GRCh38p7/known_issues/) were excluded, as well as 50 bp regions flanking indels in this list. In all the three lists above (i–iii) we used the union of the newer dbSNP150 and an older version (147 or 146), as we found some false-positive detections to be missing from the newer dbSNP version. (iv) repeatMakser-annotated rDNA repeats were excluded, as there are multiple nearly- identical such genomic regions. Therefore, reads aligned to these regions are prone to misalignments. In addition, repeatMakser-annotated *Alu* regions were excluded. In contrary to the aforementioned regions that may be prone to false-positive detections, *Alu* elements are actually known to be heavily edited^44,103^, and were excluded to prevent a bias in the accuracy calibration of the pipeline due to the numerous editing events in these regions.

Original dbSNP tables, RefSeq and repeatMakser annotations were downloaded from the UCSC genome browser website^100^(http://hgdownload.soe.ucsc.edu/goldenPath/hg38/database/). The RefSeq and the repeatMasker tables are updated as of August 8, 2017 and January 1, 2018, respectively. These filtered regions sum up to 2.1% out of the RefSeq annotation that was used.

### BLAT-based mismatches filtration

Many mismatches result from erroneous mapping of reads to a highly similar locus in the reference genome. To clean these, we used a sliding window approach, looking each time at a 76 bp-long subsequence (to match reads’ length) of all RefSeq coding sequences with a step of 19 bp (25% of the read length). These 76 bp-long subsequences were aligned to the reference genome using BLAT (BLAST-like Alignment Tool)^104^, in the search of highly-similar genomic regions (alignment length ≥ 61 bp, ≥ 94% identity, no indels in query), mainly due to duplicated genomic regions and processed pseudogenes. Mismatches found by these BLAT alignments, are likely to appear as mismatches between RNA-seq data and the reference genome, due to misalignments of sequencing reads originating from one genomic locus to another, highly-similar, locus. Thus, these mismatches were discarded from the above list of potential editing sites. A-to-G mismatches were detected in ~0.5% of all adenosines in the RefSeq annotation.

### Statistical model and score calculation

To decide whether a specific genomic position is an editing site, we employed a statistical model that integrates the data from all donors for each tissue, using a maximum likelihood approach. For each donor, three binomial tests and three *p*-values are evaluated per site, to assess the likelihood of the observed total coverage $C$ and number of reads supporting a mismatch $E$, assuming editing does not occur at this site:

1. The probability that the retrieved $C$ and $E$ are merely due to sequencing errors, given a maximal probability of base calling sequencing error of ${E}_{{{{{{\rm{error}}}}}}}={10}^{-3}$ (as set by the minimal Phred quality score to call substitution of *q* = 30):
  1 $${P}_{{{\mbox{error}}}}\left(X\le E\right)=\mathop{\sum }\limits_{j=0}^{E}\left(\begin{array}{c}{C}\\ {j}\end{array}\right){{{E}_{{{\mbox{error}}}}}^{C-j}\left({1-E}_{{{\mbox{error}}}}\right)}^{j}$$
2. The probability that the retrieved $C$ and $E$ represent a rare heterozygous SNP, given an expected frequency of the alternative allele ${E}_{{{\mbox{hetero}}}}=0.5$ and an a-priori probability of such a genomic event of ${p}_{{{\mbox{hetero}}}}={10}^{-4}$ :
  2 $${P}_{{{\mbox{hetero}}}}\left(X\le E\right)=\left(\mathop{\sum }\limits_{j=0}^{E}\left(\begin{array}{c}{C}\\ {j}\end{array}\right){{{E}_{{{\mbox{hetero}}}}}^{j}\left({1-E}_{{{\mbox{hetero}}}}\right)}^{C-j}\right)\cdot {p}_{{{\mbox{hetero}}}}$$
  ${p}_{{{\mbox{hetero}}}}$ was estimated based on^102^. The typical number of SNPs in the coding sequence ranges between 21.4 and 26 k for five distinct human populations. Of these, 1–4% are rare SNPs (prevalence < 0.5%). As the total length of RefSeq CDS is 34 Mbp, the probability to have a SNP at a given position can be as high as (2.6 × 10^4^/3.4 × 10^7^) × 0.04 = 3.06 × 10^−5^. As we defined rare SNPs as those with prevalence <1%, the probability could still be higher. Adopting a conservative approach, we have therefore set ${p}_{{{\mbox{hetero}}}}={10}^{-4}$.
3. The probability that the retrieved $C$ and $E$ represent a rare homozygous SNP, Given an expected frequency of the alternative allele ${E}_{{{\mbox{homo}}}}=1$ and an estimatedprobability of such genomic event of ${{p}_{{{{{\mathrm{homo}}}}}}}=({p}_{{{{{\mathrm{hetero}}}}}})^{2}={10}^{-8}$:
  3 $${P}_{{{\mbox{homo}}}}\left(X\le E\right)=\left(\mathop{\sum }\limits_{j=0}^{E}\left(\begin{array}{c}{C}\\ {j}\end{array}\right){{{E}_{{{\mbox{homo}}}}}^{j}\left({1-E}_{{{\mbox{homo}}}}\right)}^{C-j}\right)\cdot {p}_{{{\mbox{homo}}}}$$

We choose the most likely of the above three explanations and adopt the lowest of these three *p*-values. Had this been a bona fide *p*-value, one could have then incorporated the multiple *p*-values (one per each donor) into one statistic using Fisher’s method, and define a score $S$ per site based on the full data from $N$ donors to be (natural log):

4 $$S=-2\mathop{\sum }\limits_{i=1}^{N}{\log }\left({\min }\left[{{P}_{{{\mbox{error}}}}}_{i},{{P}_{{{\mbox{herero}}}}}_{i},{{P}_{{{\mbox{homo}}}}}_{i}\right]\right)$$

This statistic should then have a *χ*^2^ distribution with 2*N* degrees of freedom. However, as we allow for more than one test per site and choose the most likely possibility, the chosen *p*-value per donor is not truly a *p*-value (in the sense that the values are not uniformly distributed along the unity interval given the null hypothesis of no editing). Therefore, $S$ does not follow the *χ*^2^ distribution. Yet, our approach overestimates the true *p*-value, and estimating the likelihood of getting values as high as $S$ by the *χ*^2^ distribution would provide an underestimate of the statistical significance. Note, however, that the statistical significance obtained is only correct if one assumes the mismatches of different reads to be independent. De facto, we have found that many of the false-positive detections are due to systematic misalignments and other factors not satisfying this independence assumption. To avoid confusion and misinterpretation, we therefore chose not to assign a *p*-value to each site, but use the score only as a filtering step. Mismatch sites with a score $S < 2N$ were discarded ($2N$ being the mean of the distribution).

Another *p*-value-based filtration was aimed to eliminate several sorts of errors that are specific to one sequenced-strand. During the sequencing protocol, the original RNA fragments are reverse-transcribed into double-stranded cDNA which is then amplified. The sequencing machines sequence both strands of these cDNA fragments, regardless of the original expressed strand. Therefore, an RNA editing event should be manifested as a mismatch showing up in both strands of the cDNA. In contrast, mismatches due to technical sequencing and amplification errors, are often strand specific^22^. Thus, we have pooled the data from all samples and applied a binomial test to the reads aligned to each of the genomic strands, separately, requesting that both tests result in a significant *p*-value rejecting the possibility of sequencing errors (both *p*-values < ${{{{{\rm{exp }}}}}}\left(-N/4\right)$).

### Cluster-based filtering

Clusters of mismatch sites, i.e., multiple mismatch sites of different substitution types (e.g., A-to-G and C-to-A, etc.) at the same locus, might point to problematic read alignments (e.g., mapping of reads due to a polymorphic pseudogene to its original gene) leading to false-positive detections. To filter these, we calculated the positions of all mismatches relative to the mature mRNA sequences using Annovar (v2018Apr16; https://doc-openbio.readthedocs.io/projects/annovar/en/latest/)^105^ with RefSeq-transcripts built database. Using BEDtools cluster (v2.26.0)^106^, we joined pairs of mismatch-positions at a distance ≤100 bp apart into clusters, iteratively. Clusters containing more than one type of mismatch were discarded.

### Whole-exome-sequencing-based filtering

To further clean up our list from sites with a genomic origin, we analyzed WES data, deposited as a part of the GTEx dataset. Additionally, this analysis may also filter out candidate sites that resulted from alignment errors. A total of 609 WES samples from 602 distinct donors (mostly from whole blood specimens) were used (Supplementary Data 16). Matching WES data was available for 504 of the 548 RNA-seq donors. The other 98 unmatched WES data were used for the analysis as well. We mapped the WES data using the same pipeline applied to the RNA-seq samples, and calculated the WES-mismatch level at each of the putative editing sites. Sites with insufficient (<100) WES reads or a WES-mismatch level exceeding 0.1% (namely, the maximal expected sequencing error rate) were discarded.

### Recalculating editing levels and editing-level-based filtration

To further reduce noise in our set of sites we also excluded sites with low editing level, as sites in which a very small fraction of the transcripts were edited are assumed to have only a limited contribution to functionality. To correctly assess the per-site editing levels in each of the tissues, candidate sites from the 47 tissues were combined to a single list. Next, the total coverage and the number of reads supporting the mismatch (i.e., edited reads) were calculated from the original unfiltered BAM files using SAMtools mpileup (v1.2; http://www.htslib.org/)^101^. The reference genome was not supplied and the following options were applied: -B –Q 0 -d 10000000 -ff SECONDARY,UNMAP,DUP. The per-site-per-sample (i.e., donor in a certain tissue) coverage data were summed. Sites that did not exhibit an editing level ≥1% in at least one tissue were discarded. The resulting 1517 A-to-G sites, their annotation, and editing level per tissue, appear in Supplementary Data 2.

### Maximal editing levels

The maximal editing level was calculated separately for each site. It was defined as the maximum editing level across all tissues with coverage ≥100 reads. Sites’ maximal editing level was used in all non-tissue-specific analyses as a representative editing level value unless otherwise stated.

### RNA editing index

The editing index is defined as described in^44^ for all GTEx samples. Briefly, for a chosen set of A-to-G editing sites, mpileup data was used to determine the number of observed matched As and the number of mismatching Gs at the sites. The index is the percent of Gs out of the number of As and Gs combined. The editing index values were calculated separately for each tissue type unless stated otherwise. The T-to-C index was assessed in the same manner.

### Coverage calculation in nonhuman samples

Genomic coordinates of the sites orthologous to the 1517 human RNA editing sites in the 21 nonhuman reference genomes were found using the liftOver program (http://hgdownload.soe.ucsc.edu/admin/exe/linux.x86_64/liftOver) with default parameters and the appropriate conversion files (*.over.chain.gz files). Then, liftOver was used again to reconvert nonhuman coordinates back to the human reference genome, to verify these are reciprocal best hits, thus a common proxy for real orthologs. For each species, unmapped records, and records for which the orthologous site was not mapped back to the original human coordinates were discarded. Whenever the nucleotide at the orthologous genomic position in a given species was an adenosine, total coverage and a total number of edited reads were calculated for each biological sample, using mpileup as described in the paragraph above.

### Identification of conserved RNA editing sites

We used NCBI taxonomy (https://www.ncbi.nlm.nih.gov/taxonomy) to construct a phylogenetic tree for human and the 21 nonhuman mammals. Inner nodes exhibiting multifurcation were manually bifurcated according to UCSC genome browser 100-way-multiple-sequence-alignment-based calculated phylogenetic tree (http://hgdownload.soe.ucsc.edu/goldenPath/hg19/multiz100way/hg19.100way.scientificNames.nh). The tree was visualized using iTOL (ver. 5.6.3) online tool^107^.

In order to gain sufficient sequencing coverage and minimize multiple-testing (thus increasing statistical power), the 21 individual mammal species were grouped into 6 distinct groups as follows: nonhuman-Homininae, Cercopithecinae, non-Catarrhini primates, Glires, Laurasiatheria, and non-Boreoeutheria mammals (Supplementary Data 15). Due to their different editing pattern, samples in each group were further classified into two categories: brain and non-brain samples (Supplementary Data 13). The per-site values of total coverage and number of supporting reads were summed over all samples in each of the six groups of organisms and each category, resulting in 12 different lists. A binomial test was applied for each site, with the null hypothesis that the site is not edited in a given nonhuman group and category, and possible mismatches can be attributed to random sequencing errors (with a rate of 10^−3^, corresponding to *q* = 30). The resulted p-values were adjusted using Benjamini and Hochberg’s False Discovery Rate (FDR = 0.001) correction, for each of the 12 lists, separately. A human RNA editing site was considered conserved in a certain nonhuman group if it was detected in either brain or non-brain tissues. On the other hand, a site that was not found to be conserved and exhibited a non-significant > 0.05 FDR and a total coverage ≥ 500 in either brain or non-brain categories was considered non-conserved in this group of species (Supplementary Data 2). To determine the emergence of editing at a given site along mammalian evolution, we look for the last common ancestor (LCA) of human and the most distant mammal exhibiting RNA editing, for which the editing in the most recent outgroup (of the descendants of the LCA) is non-conserved. This LCA is considered to be the one at which the editing of a site has emerged.

To search for human-specific editing sites (Supplementary Data 14), we looked for either (i) edited human adenosines where the reconstructed nucleotide in the ancestral Hominin sequence is not an adenosine (and therefore not edited), or (ii) human sites exhibiting an editing level >2% in cerebellum and cortex that were deemed non-conserved (as defined above) in nonhuman-Homininae and in one of Cercopithecinae or non-Catarrhini primates.

### Calculating the relative frequency of synonymous and nonsynonymous editing sites

To estimate the extent to which our detected A-to-G sites are positively selected throughout evolution we used an approach that largely follows^94^. First, choosing the longest protein-forming transcript per gene from RefSeq annotation (see above), we counted CDS adenosines whose potential editing into inosines would have resulted in synonymous and nonsynonymous changes (*S* = 2,512,451 and *N* = 6,185,931, respectively). Then, we calculated the ratios of the number of actual editing sites in our list to the number of potential sites, resulting in *f*_S_=1.83 × 10^−4^ and *f*_N_ = 1.71 × 10^−4^ for synonymous and nonsynonymous editing, respectively. As weak editing sites are expected to have a lesser functional impact, we have repeated the calculation for weak (<10%) and strong (≥10%) sites separately. In addition, for each of the four sets of weak and strong, synonymous and non-synonymous, sites, we constructed 10^6^ sets of 1517 randomly chosen CDS adenosines, with the same distribution over chromosomes and sequence contexts (1 bp downstream and upstream), and calculated the mean and SD over the 10^6^ random replications. *P*-values were estimated by a randomization test, checking what proportion of the 10^6^ random sets produce a value equal or higher than the one measured in the actual data. Finally, we repeated the analysis, taking into account only the most strongly edited site per gene.

### Ancestral state reconstruction for RNA editing sites and classification to restorative and diversifying

Ancestral state reconstruction was performed similarly to^95^ using sequences of the following organisms: *Homo sapiens*, *Pan troglodytes*, *Pan paniscus*, *Gorilla gorilla*, *Macaca mulatta*, *Macaca fascicularis*, *Papio anubis*, *Callithrix jacchus*, *Saimiri boliviensis*, *Mus musculus* and *Bos taurus* while the latter two served as outgroups (Supplementary Fig. 6). Human RefSeq accession numbers of each gene that harbors one or more editing sites were converted to Ensembl IDs via Ensembl API (http://www.ensembl.org/info/docs/index.html), retaining only genes having one-to-one orthologous genes in all the indicated species. Next, mRNA and protein sequences of the Ensembl canonical transcript were retrieved for each ortholog. Clustal Omega (Ver. 1.2.1)^108^ with default parameters was then used to generate a per-gene multiple sequence alignment of the orthologous protein sequences. Next, the protein sequence alignments were converted to the corresponding codon alignments using PAL2NAL^109^, and these were then used to reconstruct the ancestral transcripts sequences in a maximum-likelihood-based analysis utilizing the codeml program from the PAML4 package^110^ with default parameters except for the RateAncestor = 1 option, to execute the ancestral state reconstruction process. A phylogenetic tree that includes the listed species was extracted from the more extensive tree (See above and Fig. 5) with a conventional trifurcation in its root to designate an unrooted tree (Supplementary Fig. 6). The ancestral coding sequences were evaluated for the LCA of the above-specified primates, as well as for all other intermediate ancestors between human and the LCA. The ancestral nucleotides for each editing site were retrieved from the joint reconstruction multiple sequence alignment of these sequences.

Next, based on their translated amino acids, A-to-G editing sites were classified to (1) synonymous sites where the RNA editing does not alter the translated amino acid, (2) nonsynonymous restorative sites where the amino-acid encoded by the edited human transcript appears at the same position in one or more of the ancestral protein sequences, and (3) nonsynonymous diversifying sites where editing of the human sequence generates a novel amino acid, that does not appear (at the same position) in any of the ancestral protein sequences. A subset of 828 sites were classified in total.

The same methodology was applied to all genes in the RefSeq annotation (see above) regarding all adenosines as putative RNA editing sites in order to assess the frequencies of restorative, diversifying, and synonymous sites relative to their genomic background. Additionally, 10^6^ sets of 828 randomly-selected sites were chosen out from the sites in the genomic background with the same distribution over sequence contexts (1 bp downstream and upstream), as a control. Randomization-based *p*-values were evaluated using these sets.

### Strand-specific RNA-seq validation

To show that T-to-C substitutions detected by our pipeline are mostly due to ADAR-associated A-to-I editing of transcripts expressed from the strand opposing the coding RefSeq strand, we analyzed four additional datasets of strand-specific human RNA-seq samples^36^. One dataset included 18 samples from 6 human tissues (brain, liver, lung, muscle, heart, and kidney) (SRA accession number: SRP058632). The second included 126 retina samples^111^ (all healthy samples from accession number: GSE115828, excluding SRR7461061 which failed our quality checks). The third is composed of 143 fibroblast cell line samples^112^ (accession: GSE113957).

The fourth dataset was created as follows: matched Genomic DNA and Total RNA samples of peripheral blood leukocytes from three adult male humans were obtained from AMSBIO (D1234148 and R1234148-10). Whole exome library was created with the genomic DNA using the Agilent SureSelect XT Human All Exon V6 + UTR protocol. Stranded mRNA library was created with the total RNA using illumina TruSeq Stranded protocol with polyA enrichment. Libraries were sequenced by Macrogen Europe as 150 bp paired end reads using Illumina NovaSeq6000. These newly generated blood sequencing data were deposited to SRA (BioProject ID: PRJNA715360).

These four datasets were processed as described above (Reads Alignment and BAM processing). Total coverage and the number of reads supporting the pipeline-detected A-to-G and T-to-C substitutions were calculated using mpileup, as previously mentioned. Following calculation of per-strand coverage and number of reads supporting the substitution, we applied binomial test (for each strand separately) to test whether the mismatch-harboring reads may be attributed to random sequencing errors (with a rate of 10^−3^, corresponding to *q* = 30). For each mismatch type and strand, *p*-values were adjusted using Benjamini and Hochberg’s False Discovery Rate (FDR = 0.05) correction. In addition, we have calculated the (per strand) editing index (see above) over the pipeline-detected A-to-G and T-to-C substitutions.

### Dimensionality reduction analysis

Per-Sample editing levels were calculated for each detected A-to-G site with coverage ≥ 10 reads, otherwise, the editing level was considered missing. Biological samples that had more the one GTEx sample barcode were not included. Sites or samples with >80% missing values were discarded. The remaining missing values were then imputed by the mean editing level at this site over all GTEx samples. The kidney samples were removed from the analysis due to the low number of samples (*n* = 38), compared to other tissue types (*n* ≥ 69). t-SNE was performed using the Rtsne package (version 0.15; https://github.com/jkrijthe/Rtsne), with default parameters, except for *θ* = 0.25 for better accuracy. Results were plotted using the ggplot2 package (version 3.2.1).

### Editing sites classification by targeting ADAR

We used previously published ADARs overexpression (OE)^14^ (SRA accession: SRP090260; HEK293 cells) and knockout (KO)^49^ (SRA accession: SRP200481; C57BL/6 mice brains) RNA-seq datasets. Alignment of reads to the human or mouse reference genome and quantification of per site total coverage and number of edited reads were performed as described above (Reads Alignment and BAM processing; Recalculating Editing Levels and Editing-Level-Based Filtration; and Coverage calculation in nonhuman samples). As the analysis of editing in mouse was dependent on liftOver conversion, 439 sites were excluded due to a non-coherent or a non-A conversion result, leaving 1078 sites. Importantly, none of these sites have shown A-to-G mismatches at an appreciable level in ADAR double-knockout samples (Supplementary Data 13). First, biological replicates were pooled, and Fisher’s exact test was applied, comparing the numbers of edited and non-edited reads per site in each targeted ADAR and genetic modification to those in the appropriate WT control. A site was considered as enzyme-specific if its editing level was found to be significantly altered (Benjamini–Hochberg–corrected *p*-value ≤ 0.05) by either the OE or the KO for one of the ADARs but not for the other and if the editing level was changed in agreement with the nature of the modification (i.e., higher editing levels for OE and lower for KO). When significant *p*-values were obtained for both Adar1 and Adar2 the site was considered as a shared target of both enzymes.

In addition, we estimated the relative influence of Adar1 (*I*) on the editing level at site as follows:

5 $${I}_{i}=\,\frac{{{{\log }}}_{2}\left(\frac{{{{{{\rm{WT}}}}}}\,{{{{{\rm{editing}}}}}}\,{{{{{\rm{level}}}}}}\;+\;0.001}{{Adar}1\,{{{{{\rm{KO}}}}}}\,{editing}\,{{{{{\rm{level}}}}}}\;+\;0.001}\right)\,}{\,{{{\log }}}_{2}\left(\frac{{{{{{\rm{WT}}}}}}\,{{{{{\rm{editing}}}}}}\,{{{{{\rm{level}}}}}}\;+\;0.001}{{{{{{\rm{Adar}}}}}}1\,{{{{{\rm{KO}}}}}}\,{{{{{\rm{editing}}}}}}\,{{{{{\rm{level}}}}}}\;+\;0.001}\right)\,+\,{{{\log }}}_{2}\left(\frac{{{{{{\rm{WT}}}}}}\,{{{{{\rm{editing}}}}}}\,{{{{{\rm{level}}}}}}\;+\;0.001}{{{{{{\rm{Adar}}}}}}2\,{{{{{\rm{KO}}}}}}\,{{{{{\rm{editing}}}}}}\,{{{{{\rm{levels}}}}}}\;+\;0.001}\right)\,}$$

Negative values of *I*_*i*_ were replaced by *I*_*i*_ = 0.

### Identification of ECS

To search for complementary sequences, we retrieved a 41bp-long genomic sequence flanking each of the editing sites (20 bp in each side) and used AB_BLAST (release: 2020-03-17; https://www.advbiocomp.com/blast.html) to look for a reversely-oriented complementary sequence within 20,001 bp (10k in each side) surrounding the editing site (command: ab-blastn *W* = 4 *Q* = 14 *R* = 4 -matrix=RNA hspmax=5). Hits of length ≥30 and identity ≥70%, residing within the same transcript or up to 5 kb downstream to it (accounting for long, unannotated UTRs) were considered as putative ECS. As a control, we randomly chose 1517 exonic adenosines that are not known to be edited. For both editing and control sites, minimum free energy (Δ*G*) was calculated for the longest BLAST hit (within the transcript or 5000 bp downstream, but regardless of its length and identity) applying the fold program from the RNAStructure package^113^ to the query region and the subject region of the hit, connected with a 100 bp poly(A) linker when needed. Sites for which either the calculated free energy was positive or no hit was found were assigned Δ*G* = 0.

### Multi-species RNA secondary structure comparisons

Multi-species dsRNA structures were calculated for regions within 3 genes: ASNS, NEIL1 and SEMA5B. Human ECS were detected as mentioned above with the exception of SEMA5B, where the BLAST-dependent analysis was not sensitive enough to detect the ECS. Instead, a 72 bp-long region that harbors the single editing site in the coding sequence of SEMA5B and found to form a dsRNA structure *in-silico* was used. Next, nonhuman homologous sequences of the edited region and ECS were retrieved, using the UCSC multiz30way (ASNS and SEMA5B) and multiz100way (NEIL1) tables. RNA secondary structures were predicted, for each gene separately, using the RNAStructure Multilign program with default parameters. Sequences from species with non-editable CDS variants were folded separately from editable ones. Next, to determine the position of mismatches between the homologous sequence we used Clustal Omega (Ver. 1.2.4)^108^ program to generate their multiple sequence alignment.

### Proteomic evidence for editing

In order to detect editing events at the protein level we downloaded mass spectrometry data, available at the PRIDE database^40^. We analyzed 311 proteomic and phosphoproteomic samples, (Supplementary Data 4) originated from various human tissues, digested using trypsin, and quantified using label-free quantification (LFQ), tandem mass tag (TMT), or isobaric tag for relative and absolute quantification (iTRAQ).

To prepare a database of all possible peptides, including the ones derived from the 1036 nonsynonymous RNA editing sites in our set, we used RefSeq transcripts and considered all possible combinations of edited and non-edited states at all A-to-G editing sites detected by our pipeline. The translated transcripts were in silico trypsin-digested into peptides, taking into account up to two miss-cleavages per peptide. We considered potential trypsin cleavages losses and gains due to editing but not stop losses. In addition to this set of peptides, we created two additional peptide databases as controls: (i) a proteome where RefSeq transcripts may be “edited” at the 851 nonsynonymous non-A-to-G sites detected by our pipeline (sites we consider as noise). Some of these are likely to be found in the proteome, as they represent genomic variability or misalignment due to genomic duplications. (ii) A proteome where RefSeq transcripts may be “edited” at 1036 randomly-chosen genomic positions to create a nonsynonymous change. These random sites were matched to the 1036 detected recoding sites in terms of the edited codon, and (if possible) were chosen within the same exon as the true edited site (829 sites). In cases an identical codon was not found in the same exon, we picked a position in the same codon from another exon of the same gene (187 sites) if possible, or in other genes otherwise (20 sites).

The proteomic samples were compared to this set of potential peptides using MaxQuant (MQ)^114^, with default parameters and several modifications, as follows: for phosphoproteome samples we added phosphor-STY modification to the variable modifications list; LFQ samples were analyzed with the parameter lfqmode=1 for delayed normalization using MQ LFQ algorithm; for TMT and for iTRAQ samples we chose 11plex-TMT and 8plex-iTRAQ in the isobaric labels parameters section, respectively (with reporter ion MS2). Peptides that may have derived from common contaminants (based on MQ Contaminants Fasta file) were excluded. We then considered only peptides that are distinct to either the non-edited or the edited versions. For each editing site and each sample, we recorded peptides that support the genomic version, the edited version, or both. The same search was applied to the two control databases.

### Estimating biological variability of editing levels

To assess the variability of editing across donors, we first summed the total coverage and the number of edited reads over a number of tissues. To avoid variability due to a different set of available tissue samples, we wanted to have the exact same set of tissues for all donors considered. Since not all tissues are available for all donors, we had to choose a subset of donors and a subset of tissues, trying to keep both the number of donors and the number of brain regions sufficiently large to gain statistical power. Focusing on the brain, we studied 65 donors for each of which we pooled the coverage and editing data from five brain regions: caudate, nucleus accumbent, cerebellum, cerebellar hemisphere, and putamen.

Next, we used the following model to separate the stochastic sampling noise and estimate the biological variability across samples. For each editing site, denote ${c}_{i}$ and ${g}_{i}\,$the total coverage and number of edited reads in sample $i$, respectively. The observed editing level per sample is therefore ${e}_{i}={g}_{i}/{c}_{i}$. We assume ${g}_{i}$ to be distributed binomially ${g}_{i} \sim B\left({{c}_{i},p}_{i}\right)$, where ${p}_{i}\,$is the underlying editing level per site per sample (i.e., fraction of edited cDNA molecules). This level may be different from sample to sample, and we denote its mean and variance over the population by $p,{v}$, respectively. The mean observed level is thus

6 $${\left\langle {e}_{i}\right\rangle }_{S,B}={\left\langle \frac{{g}_{i}}{{c}_{i}}\right\rangle }_{S,B}={\left\langle {p}_{i}\right\rangle }_{S}=p$$

where ${\left\langle \ldots \right\rangle }_{S,B}$ denotes averaging over both samples (*S*) and the binomial distribution (*B*), and we have used the relation ${\left\langle {g}_{i}\right\rangle }_{B}={p}_{i}{c}_{i}$. The variance of the observed editing level (over both samples and binomial distributions) is

7 $${{{{{\rm{Var}}}}}}\left({e}_{i}\right) 	={\left\langle {{e}_{i}}^{2}\right\rangle }_{S,B}-{{\left\langle {e}_{i}\right\rangle }_{S,B}}^{\!}{2}={\left\langle \frac{{\left\langle {{g}_{i}}^{2}\right\rangle }_{B}}{{{c}_{i}}^{2}}\right\rangle }_{S}-{p}^{2}\\ 	={\left\langle \frac{1}{{{c}_{i}}^{2}}\left({{c}_{i}}^{2}{{p}_{i}}^{2}+{c}_{i}{p}_{i}\left(1-{p}_{i}\right)\right)\right\rangle }_{S}-{p}^{2}=\,{\left\langle {{p}_{i}}^{2}\right\rangle }_{S}-{p}^{2}+{\left\langle \frac{1}{{c}_{i}}{p}_{i}\left(1-{p}_{i}\right)\right\rangle }_{S}\\ 	 =v+{\left\langle \frac{1}{{c}_{i}}\right\rangle }_{S}{\left\langle {p}_{i}\left(1-{p}_{i}\right)\right\rangle }_{S}=v\left(1-{c}^{-1}\right)+{c}^{-1}\left(p-{p}^{2}\right)$$

where ${c}^{-1}={\left\langle \frac{1}{{c}_{i}}\right\rangle }_{S}$ and the penultimate equation assumes that coverage and editing level distributions over samples are uncorrelated. One thus obtains the following for the variance over the population of the underlying editing level:

8 $$v=\frac{{{{{{\rm{Var}}}}}}\left({e}_{i}\right)-{c}^{-1}\left(p-{p}^{2}\right)}{1-{c}^{-1}}$$

For a given set of samples, one may estimate p and ${c}^{-1}$ as the mean (over samples) of ${e}_{i}$ and $\frac{1}{{c}_{i}}$, respectively, and then apply the above formula to obtain an estimate $v$. Note that this estimate may occasionally turn out negative, due to statistical errors in these estimates. In these cases, our best estimate for the variability in the underlying editing level is zero. Finally, we point out that some of the variability across samples of the underlying editing level may be due to technical rather than biological reasons (sample-to-sample differences in the sequencing protocol that may have an effect on the variance). The above estimate is therefore an over-estimate of the biological variability.

### Differentially edited sites in cancer and cardiovascular diseases

To look for differential editing in cancer, RNA-seq samples for tumor samples and matching controls were downloaded from The Cancer Genome Atlas^62^. Following^59^, we considered the nine cancer types for which healthy controls are available. Reads were re-aligned to the genome and editing levels were calculated per sample per site, as described above (Reads Alignment and BAM processing; and Recalculating Editing Levels and Editing-Level-Based Filtration).

For each donor, we excluded sites for which the number of reads aligned in the normal and tumor samples combined was <40. Then, we calculated the editing level in the pooled normal samples and the pooled tumor samples, separately, and excluded sites for which both editing levels were <0.5% in any of the cancer types. Paired-samples student’s t-test was then applied to the remaining sites, for each tissue type, to compare the editing levels per sample between the matched normal and tumor groups, followed by Benjamini–Hochberg multiple testing correction (FDR = 0.05). We further requested an appreciable >10% difference in the mean editing level between the groups.

Similarly, we looked at the editing levels observed in the three artery tissue types available in GTEx (aorta, coronaries, and tibial). For each tissue and site, we looked at the 27 diseases and conditions annotated in GTEx. We discarded low-coverage donors (<20 reads), and applied Mann–Whitney *U* test to compare editing level per site between healthy and diseased subjects, provided that both groups included at least 10 subjects. Benjamini–Hochberg FDR correction was applied (FDR = 0.05) per-disease per-tissue, and we further requested an appreciable >10% difference in the mean editing level between the groups. None of the sites identified is adjacent (<2000 bp distance) to known GWAS loci.

### Single-cell differential RNA editing analysis

We used *Tabula Muris*^115^, a recently-published comprehensive mouse single-cell dataset to test for variations in editing levels between different cell populations within the same tissue. Smart-seq2 full-length RNA-seq data from 44,494 different cells (SRA accession: SRP131661) were aligned as performed for other nonhuman samples (see above). Total coverage at the editing sites and editing levels were assessed as described above (Recalculating Editing Levels and Editing-Level-Based Filtration) for the classification by targeting ADAR. Next, per-cell total coverage and editing values were summarized across all cells in each cell population based on the previously-published annotations^115^. As for lung cells, a revised and more accurate annotation was used^116^ (Supplementary Data 6). Then, sites that did not exhibit an editing level ≥10% with minimal coverage of 5 reads in at least one cell population were excluded, and for each site, all possible pairs of cell populations within the same tissue were compared to detect significant differences in editing levels. A total of 14,503 pairs were evaluated in both the cell-population and the single-cell levels. At the cell-population level, the total numbers of edited and non-edited reads for each pair were compared using Fisher’s exact test. To assess differential editing levels using single-cell data, per-cell editing levels were calculated for all sites that were considered for the analysis, and compared between different cell populations within the same tissue using Mann–Whitney *U* test. *P* values from both comparisons were adjusted using the Benjamini–Yekutieli method. Comparisons with adjusted *p* ≤ 0.05 are shown in Supplementary Data 7.

### Reporting summary

Further information on research design is available in the Nature Research Reporting Summary linked to this article.

## Supplementary information

Supplementary Information

Description of Additional Supplementary Files

Datasets 1-16

Reporting Summary

## Data Availability

The data supporting the findings of this study are available from the corresponding authors upon reasonable request. Human data used in the paper is available upon dbGaP approval (GTEx: phs000424.v8.p2; TCGA: phs000178.v11.p8). Newly generated next-generation sequencing data reported in this study have been deposited in the SRA database, BioProject ID: PRJNA715360. Non-human RNA-seq samples analyzed are available from SRA (see detailed in Supplementary Data 13). Source data are provided with this paper.

## Source data

Source Data

## Code availability

The scripts used to produce the data are available at GitHub (https://github.com/a2iediting/deNovo-Detect)^117^.
