# Supplementary material for: Landscape of adenosine-to-inosine RNA recoding across human tissues
Source: Nat Commun. 2022 Mar 4;13:1184. doi: 10.1038/s41467-022-28841-4 (PMC8897444; doi:10.1038/s41467-022-28841-4)
Supplement: Supplementary file 1 — Supplementary Information [file 41467_2022_28841_MOESM1_ESM.pdf]

Supplementary Figures

Landscape of adenosine-to-inosine RNA recoding across human tissues

Orshay Gabay[1], Yoav Shoshan[2], Eli Kopel[1], Udi Ben-Zvi[1], Tomer D. Mann[3], Noam Bressler[2], Roni Cohen-Fultheim[1], Amos Schaffer[1], Shalom Hillel Roth[1], Ziv Tzur[1], Erez Y. Levanon[1,4*], Eli Eisenberg[2*]

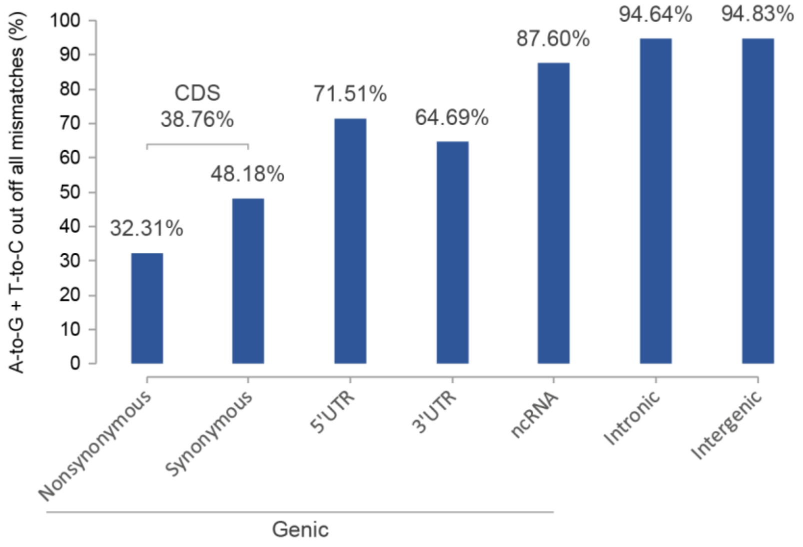

Supplementary Fig. 1: Standard editing-detection approaches perform poorly in the coding region, related to Figure 1. The relative percentage of A-to-G and T-to-C mismatches out of all mismatch types is presented for one state-of-the-art pipeline for RNA editing detection (Ramaswami et al, 2013); Repetitive non-Alu + non-repetitive; Brain, separate samples). The method exhibits high precision in intronic and intergenic regions, as demonstrated by the high percentage of A-to-G and T-to-C mismatches (among all mismatches) but fails in the coding sequence.

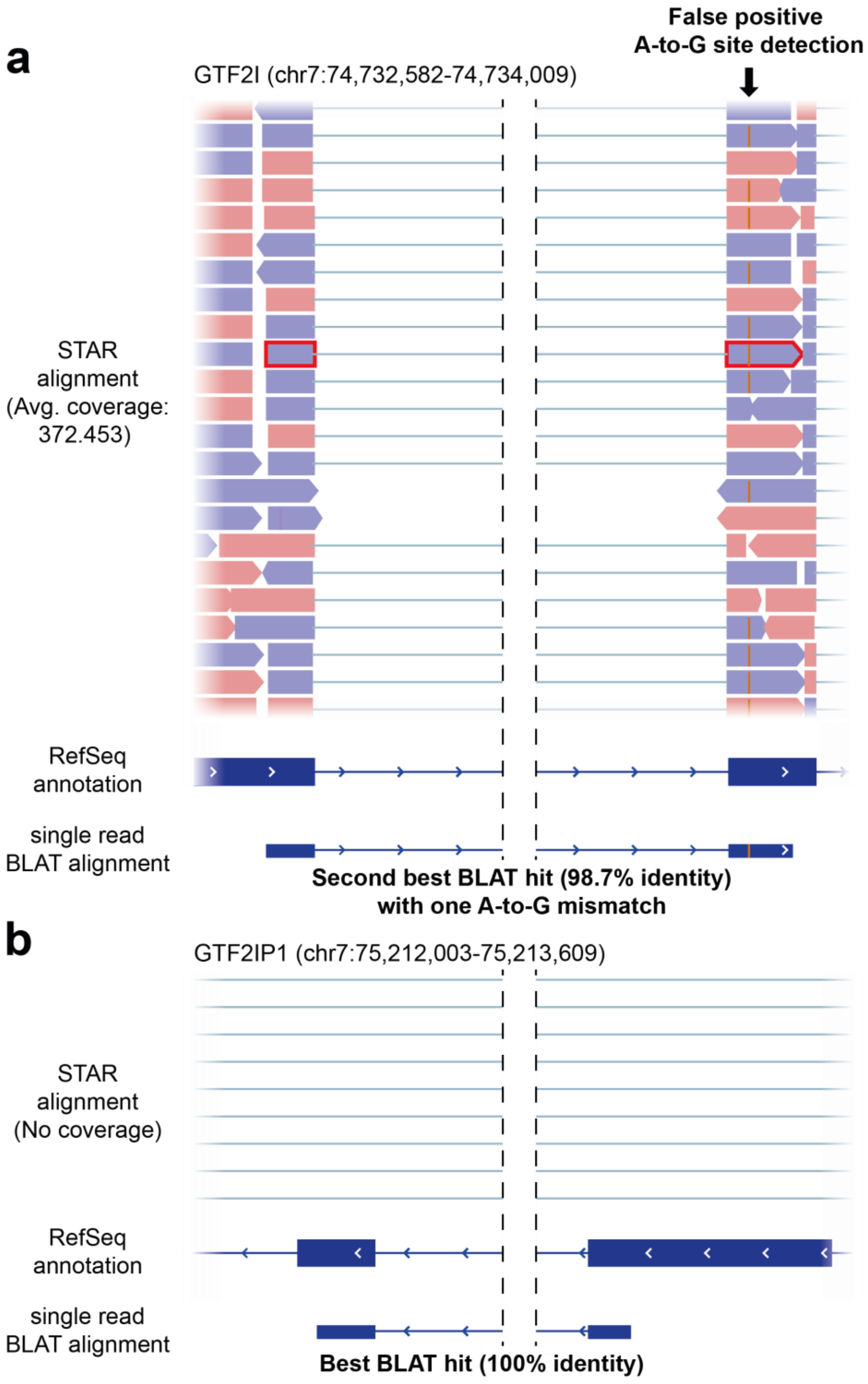

Supplementary Fig. 2: Systematic misalignments lead to apparently consistent mismatches that may be misidentified as an editing events. (a) Multiple reads aligned to the GTF2I gene by STAR aligner exhibit a consistent A-to-G mismatch. (b) However, alignment of the same reads by BLAT reveals that this mismatch-containing alignment is suboptimal, and on fact the same reads can be mapped with no mismatches to the GTF2IP1 gene.

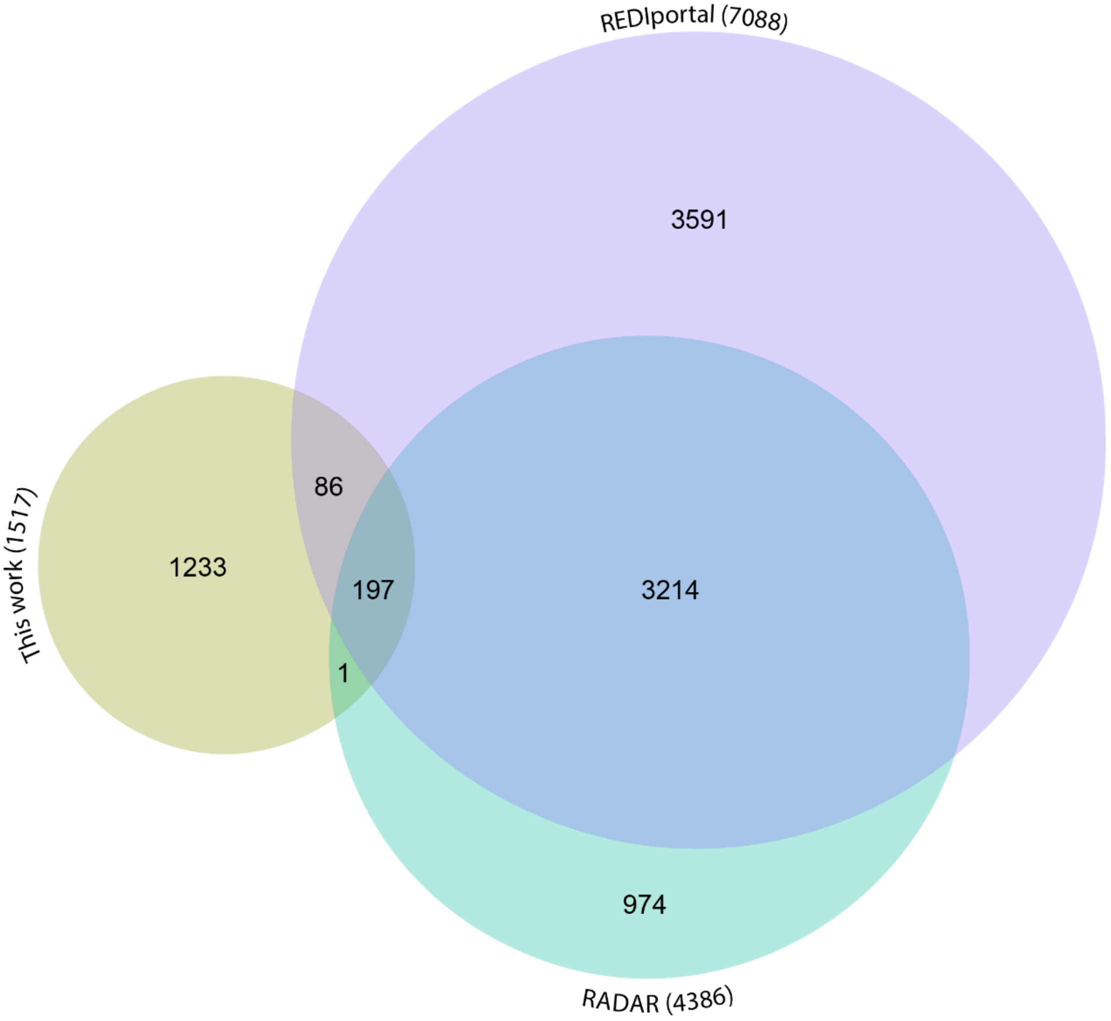

Supplementary Fig. 3: Overlap of editing sites detected in this study with non-repetitive sites in coding region reported in RADAR[13] and REDIportal[14] databases.

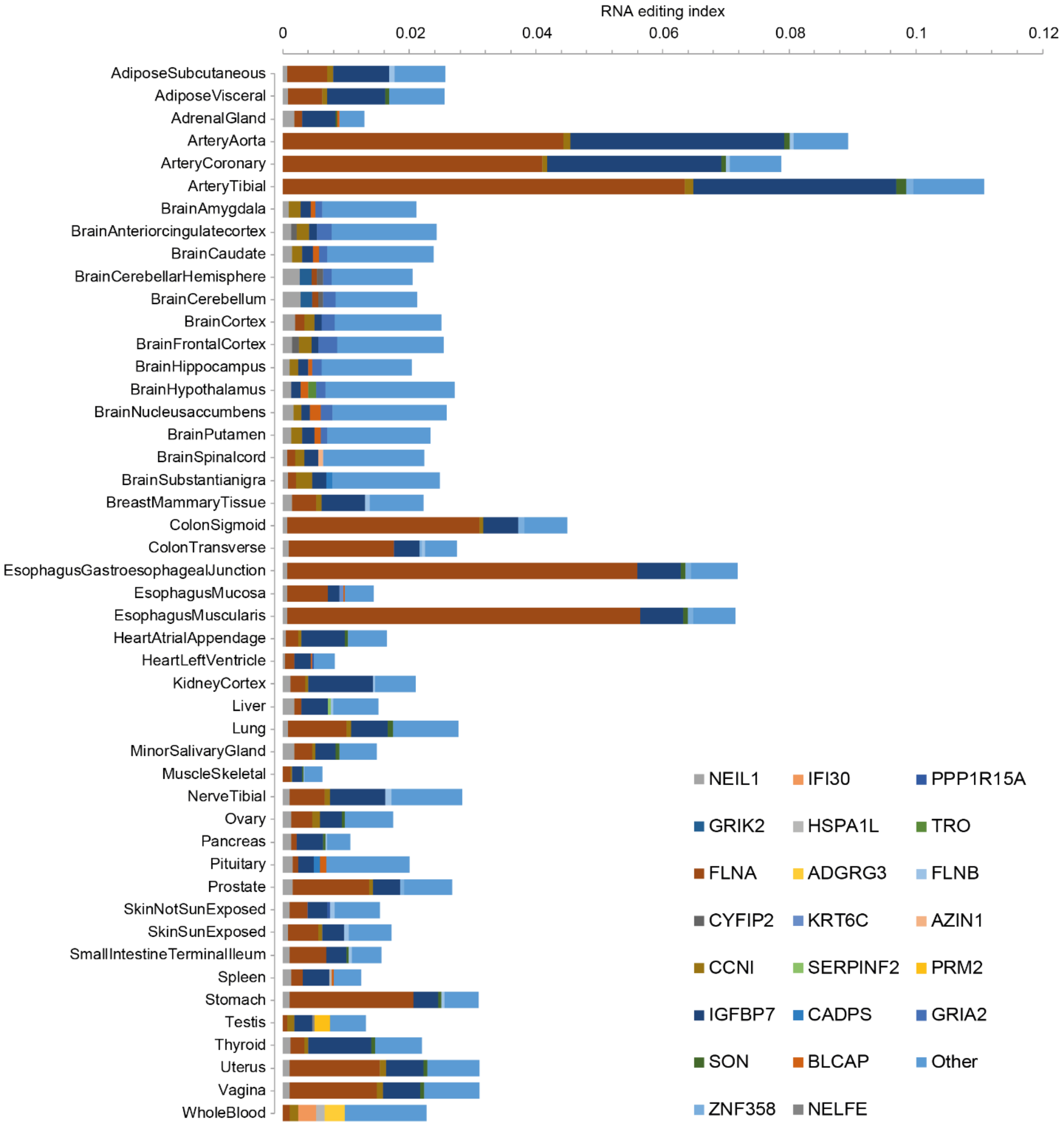

Supplementary Fig. 4: A unique editing pattern in brain regions, related to Figure 3. For each tissue-type, the bar size represents the editing index over the 1,517 CDS sites. That is, the number of edited reads (i.e. reads with a base call of G instead of A) summed over all sites, divided by the total number of reads covering these sites in the same tissue type. For each tissue type, the relative contribution of the five genes contributing the most to the index (highest number of reads showing G) is marked in distinctive colors, while all other genes were aggregated and presented as "Other". Noticeably, in most non-brain tissues the majority of the editing index is due to FLNA and IGFBP7 and the fraction reads that came from other genes is rather small. On the other

hand, brain tissues exhibit a different pattern, where the top edited genes are different, and the editing activity is widely distributed across multiple targets.

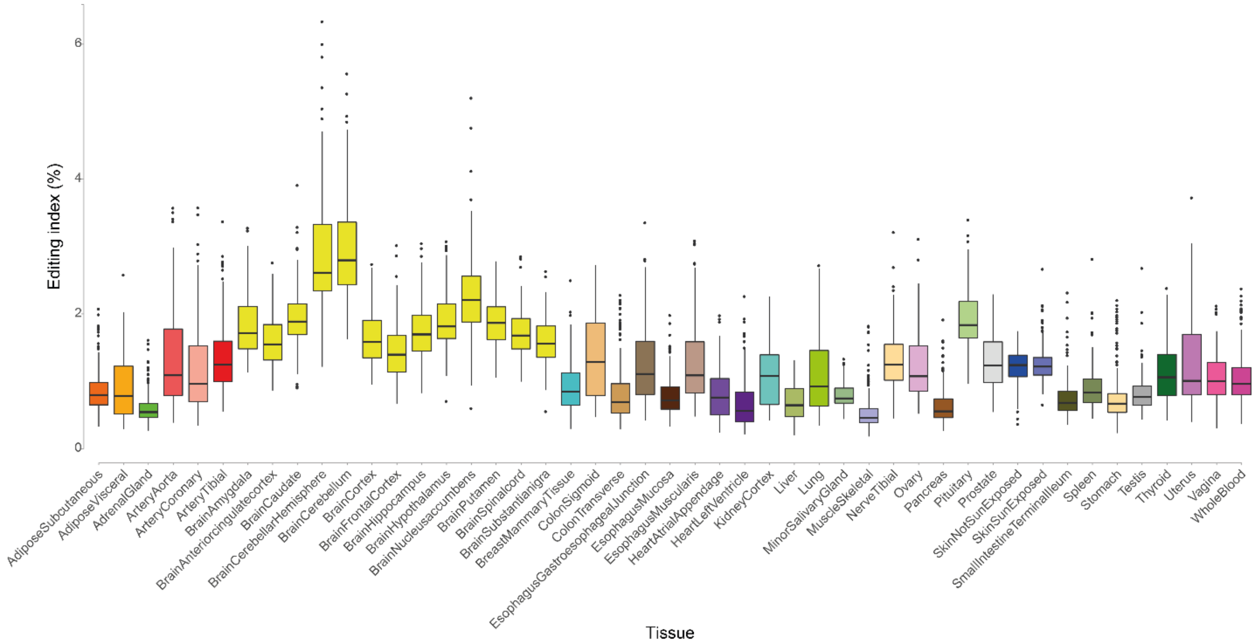

Supplementary Fig. 5: Noncoding A-to-I editing, annotated as T-to-C CDS sites, is enriched in the brain, related to Figure 4. Box-and-whisker plots, depicting the distribution of per-tissue editing-index values of the detected T-to-C sites, most of which are due to A-to-I editing of RNA molecules originating from the strand opposing an annotated coding region. Editing of these sites is elevated in brain regions, mostly in cerebellum.

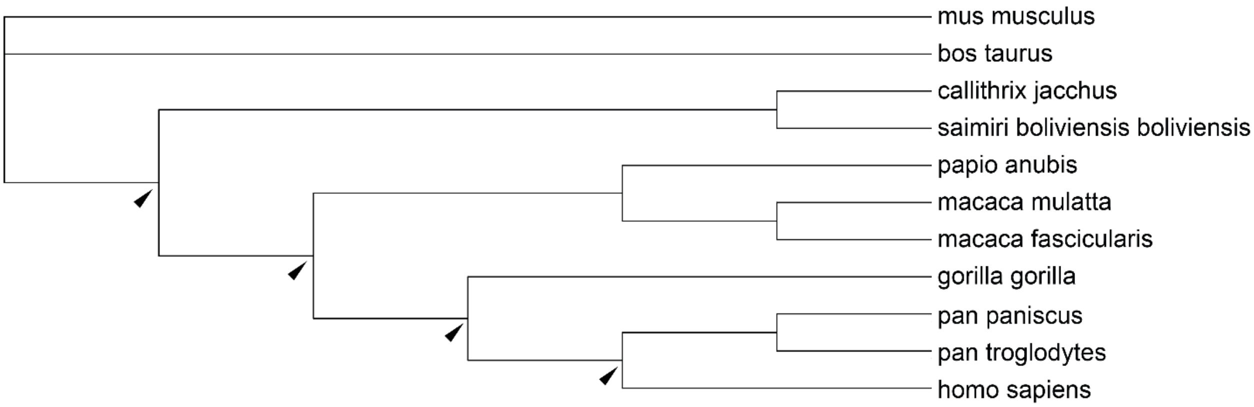

Supplementary Fig. 6: A simplified phylogenetic tree used for ancestral state reconstruction, related to Figure 5. Due to an insufficient number of genes with one-to-one orthologous in all tested organisms, we constructed the ancestral sequences based on a simplified subtree. Black arrowheads represent the 4 ancestors for which the sequences were calculated and used for classifying the sites into restorative, diversifying or synonymous. Branches are not drawn to scale.
