# Supplementary material for: Landscape of adenosine-to-inosine RNA recoding across human tissues
Source: Nat Commun. 2022 Mar 4;13:1184. doi: 10.1038/s41467-022-28841-4 (PMC8897444; doi:10.1038/s41467-022-28841-4)
Supplement: Supplementary file 2 — Description of Additional Supplementary Files [file 41467_2022_28841_MOESM2_ESM.docx]

**File: Supplemental Data 1-16**

**Supplementary Data 1** List of 9,125 GTEx RNA-seq samples with donor ID and tissue of origin

**Supplementary Data 2** Set of 1,517 CDS A-to-G site, including position, affected gene, classification, RNA-seq coverage and G calls across human tissues and in brain and non-brain in nonhuman mammals (all samples aggregated) and conservation\non-conservation status. Columns contents are explained in the table header. NA: consistent liftover conversion or conservation\non-conservation status not available. C, G or T: - designate the converted nucleotide in cases where the human adenosine was not conserved in the target nonhuman assembly.

**Supplementary Data 3** Total coverage and number of edited reads in 4 different strand-specific RNA-seq datasets for A-to-G and T-to-C sites. All samples in each dataset (separately) were pooled for the sense and antisense strands.

**Supplementary Data 4** List of mass spectrometry proteomic samples downloaded from the PRIDE database with number of AG, non-AG and random AG hits pre-sample

**Supplementary Data 5** Per-site detection of edited and non edited peptides in proteomic data

**Supplementary Data 6** Total RNA-seq coverage and G calls at A-to-G sites across 170 different cell populations from 20 mouse tissues. Per-population coverage values were obtain by summing up coverage information of RNA-seq data from 44,949 individual cells. The list include 415 sites that showed a minimum of 1% editing and 5 covering reads in at least one cell population.

**Supplementary Data 7** Significant comparisons (either at the cell-population level or at the single-cell level) of differential RNA editing levels between cells or cells populations from the same tissues. Only sites with at least 10% editing in one population or more were considered. Editing levels for cell populations or for single cells were evaluated only for sites with coverage of at least 5 reads.

**Supplementary Data 8** ADAR OE HEK293 strains classification analysis results, including per site A and G calls for each sample, per strain editing levels and ADARs classification

**Supplementary Data 9** ADAR KO mice strains classification analysis results, including per site A and G calls for each sample, per strain editing levels and ADARs classification

**Supplementary Data 10** Variability across samples and estimation of biological variance

**Supplementary Data 11** Differential CDS editing between healthy and cancerous tissues in the TCGA dataset. For each site (row), the results are shown for each of the 9 cancer types for which paired (healthy-cancer) samples where present in the TCGA (columns). Eight A-to-G sites from our set show a significant and appreciable (>10%) change of their editing level in at least one of the nine cancer types studied. For each site and tissue, the following details are listed - total aligned reads for the healthy and ill sample, summed editing index for the healthy and ill sample, p-value for the presence of editing in the ill and healthy tissues and p-values for the presence of differential editing between the editing index of individual samples. The p-values for presence of editing in healthy or ill samples is the output of a one-sided binomial test against the null hypothesis of the editing level is equal or less then 0.1%. The p-value for the presence of differential editing is the output of a two-sided students t-test against the null hypothesis that the mean editing index across the tissue samples is equal between healthy and ill samples. All p-values are FDR corrected for multiple testing. Empty cells represent site-tissue pairs that didn't pass the filtration criteria.

**Supplementary Data 12** Pneumonia-infected individuals versus controls differentially-edited sites in the arteries

**Supplementary Data 13** List of 5,673 nonhuman mammalian samples with tissue and body-parts grouping information, reference to publication and relevant accession numbers

**Supplementary Data 14** List of 20 human-specific RNA editing sites. In most cases the reconstructed ancestral Hominini sequence was non-A or unavailable, so editing was not possible. In three cases the ancestral sequence is A but the editing itself was not conserved in non-human mammals.

**Supplementary Data 15** List of organisms and reference genome assemblies used in this work, with grouping to 6 conservation groups

**Supplementary Data 16** List of 609 GTEx WES samples with donor ID and tissue of origin
