# Supplementary material for: Landscape of adenosine-to-inosine RNA recoding across human tissues
Source: Nat Commun. 2022 Mar 4;13:1184. doi: 10.1038/s41467-022-28841-4 (PMC8897444; doi:10.1038/s41467-022-28841-4)
Supplement: Supplementary file 4 — Reporting Summary [file 41467_2022_28841_MOESM4_ESM.pdf]

nature portfolio

Corresponding author(s): Erez Y. Levanon and Eli Eisenberg

Last updated by author(s): Dec. 19, 2021

Reporting Summary

Nature Portfolio wishes to improve the reproducibility of the work that we publish. This form provides structure for consistency and transparency in reporting. For further information on Nature Portfolio policies, see our Editorial Policies and the Editorial Policy Checklist.

Statistics

For all statistical analyses, confirm that the following items are present in the figure legend, table legend, main text, or Methods section.

n/a	Confirmed	
☐	☒	The exact sample size (*n*) for each experimental group/condition, given as a discrete number and unit of measurement
☐	☒	A statement on whether measurements were taken from distinct samples or whether the same sample was measured repeatedly
☐	☒	The statistical test(s) used AND whether they are one- or two-sided<br>*Only common tests should be described solely by name; describe more complex techniques in the Methods section.*
☒	☐	A description of all covariates tested
☐	☒	A description of any assumptions or corrections, such as tests of normality and adjustment for multiple comparisons
☐	☒	A full description of the statistical parameters including central tendency (e.g. means) or other basic estimates (e.g. regression coefficient) AND variation (e.g. standard deviation) or associated estimates of uncertainty (e.g. confidence intervals)
☐	☒	For null hypothesis testing, the test statistic (e.g. *F*, *t*, *r*) with confidence intervals, effect sizes, degrees of freedom and *P* value noted<br>*Give P values as exact values whenever suitable.*
☒	☐	For Bayesian analysis, information on the choice of priors and Markov chain Monte Carlo settings
☒	☐	For hierarchical and complex designs, identification of the appropriate level for tests and full reporting of outcomes
☒	☐	Estimates of effect sizes (e.g. Cohen's *d*, Pearson's *r*), indicating how they were calculated

Our web collection on statistics for biologists contains articles on many of the points above.

Software and code

Policy information about availability of computer code

Data collection	SRA Toolkit (v2.8.0)<br>STAR RNA-seq aligner (vSTAR_2.5.2b)<br>Picard Tools MarkDuplicates program (v2.6.0)<br>BamUtil clipOverlap (v1.0.13)<br>SAMtools marge (v0.1.18)
Data analysis	Annovar (v2018Apr16)<br>BEDtools cluster (v2.26.0)<br>SAMtools mpileup (v1.2)<br>liftOver program<br>iTOL (ver. 5.6.3)<br>PAL2NAL (v14)<br>codeml program from the PAML4 package (v4.9)<br>Rtsne package (version 0.15)<br>AB_BLAST (release: 2020-03-17)<br>RNAStructure package (v6.1)<br>Clustal Omega (v1.2.1 and v1.2.4)<br>MaxQuant (v1.6.10.43)<br>BLAT (BLAST-like Alignment Tool)

For manuscripts utilizing custom algorithms or software that are central to the research but not yet described in published literature, software must be made available to editors and reviewers. We strongly encourage code deposition in a community repository (e.g. GitHub). See the Nature Portfolio guidelines for submitting code & software for further information.

Data

Policy information about availability of data

All manuscripts must include a data availability statement. This statement should provide the following information, where applicable:

- Accession codes, unique identifiers, or web links for publicly available datasets
- A description of any restrictions on data availability
- For clinical datasets or third party data, please ensure that the statement adheres to our policy

We analyzed publicly available human RNA-seq data from the Genotype-Tissue Expression (GTEx) project (dbGaP Study Accession: phs000424.v8.p2) and The Cancer Genome Atlas (dbGaP Study Accession: phs000178.v11.p8), as well as thousands of publicly available non-human samples, as detailed in Supplementary Data 13.
Newly generated next-generation sequencing data reported in this study are available at SRA. BioProject ID: PRJNA715360. The scripts used to produce the data can be found at GitHub (https://github.com/a2iediting/deNovo-Detect).

Field-specific reporting

Please select the one below that is the best fit for your research. If you are not sure, read the appropriate sections before making your selection.

☒ Life sciences ☐ Behavioural & social sciences ☐ Ecological, evolutionary & environmental sciences

For a reference copy of the document with all sections, see nature.com/documents/nr-reporting-summary-flat.pdf

Life sciences study design

All studies must disclose on these points even when the disclosure is negative.

Sample size	We used all the GTEx and TCGA data available. No sample size calculation was needed.
Data exclusions	No data exclusion.
Replication	As this study analyzed existing data, no experimental replication was applied.
Randomization	Randomization is not relevant, as there are no multiple experimental groups in the study (except for grouping by species, tissue of origin, or disease state, which was done based on the annotation supplied by the databases used).
Blinding	There was no group allocation beyond the one based on species, tissue of origin, or disease state. The different groups were thus pre-determined by the annotation provided in the datasets analyzed. Thus, we were not blind to allocation at the analysis stage.

Reporting for specific materials, systems and methods

We require information from authors about some types of materials, experimental systems and methods used in many studies. Here, indicate whether each material, system or method listed is relevant to your study. If you are not sure if a list item applies to your research, read the appropriate section before selecting a response.

Materials & experimental systems

n/a	Involved in the study	
☒	☐	Antibodies
☒	☐	Eukaryotic cell lines
☒	☐	Palaeontology and archaeology
☒	☐	Animals and other organisms
☒	☐	Human research participants
☒	☐	Clinical data
☒	☐	Dual use research of concern

Methods

n/a	Involved in the study	
☒	☐	ChIP-seq
☒	☐	Flow cytometry
☒	☐	MRI-based neuroimaging
